# Quantum deep reinforcement learning for clinical decision support in oncology: application to adaptive radiotherapy

**DOI:** 10.1038/s41598-021-02910-y

**Published:** 2021-12-07

**Authors:** Dipesh Niraula, Jamalina Jamaluddin, Martha M. Matuszak, Randall K. Ten Haken, Issam El Naqa

**Affiliations:** 1 Department of Machine Learning, H. Lee Moffitt Cancer Center and Research Institute, Tampa, FL 33612 USA; 2 Department of Nuclear Engineering and Radiological Sciences, University of Michigan, Ann Arbor, MI 48109 USA; 3 Department of Radiation Oncology, University of Michigan, Ann Arbor, MI 48109 USA

**Keywords:** Non-small-cell lung cancer, Quantum information, Qubits, Quantum simulation, Translational research, Machine learning, Cancer, Health care

## Abstract

Subtle differences in a patient’s genetics and physiology may alter radiotherapy (RT) treatment responses, motivating the need for a more personalized treatment plan. Accordingly, we have developed a novel quantum deep reinforcement learning (qDRL) framework for clinical decision support that can estimate an individual patient’s dose response mid-treatment and recommend an optimal dose adjustment. Our framework considers patients’ specific information including biological, physical, genetic, clinical, and dosimetric factors. Recognizing that physicians must make decisions amidst uncertainty in RT treatment outcomes, we employed indeterministic quantum states to represent human decision making in a real-life scenario. We paired quantum decision states with a model-based deep q-learning algorithm to optimize the clinical decision-making process in RT. We trained our proposed qDRL framework on an institutional dataset of 67 stage III non-small cell lung cancer (NSCLC) patients treated on prospective adaptive protocols and independently validated our framework in an external multi-institutional dataset of 174 NSCLC patients. For a comprehensive evaluation, we compared three frameworks: DRL, qDRL trained in a Qiskit quantum computing simulator, and qDRL trained in an IBM quantum computer. Two metrics were considered to evaluate our framework: (1) similarity score, defined as the root mean square error between retrospective clinical decisions and the AI recommendations, and (2) self-evaluation scheme that compares retrospective clinical decisions and AI recommendations based on the improvement in the observed clinical outcomes. Our analysis shows that our framework, which takes into consideration individual patient dose response in its decision-making, can potentially improve clinical RT decision-making by at least about 10% compared to unaided clinical practice. Further validation of our novel quantitative approach in a prospective study will provide a necessary framework for improving the standard of care in personalized RT.

## Introduction

The efficacy of radiotherapy (RT), as one of the main clinical cancer treatments, has the potential to be improved by personalization according to each patient’s estimated treatment response. In a study by Bryant et al., it was estimated that about 3.05 million cancer survivors in 2016 were treated with radiation, accounting for 29% of all cancer survivors. The number of radiation-treated cancer survivors was expected to be 3.38 million by 2020 and is projected to be 4.17 million by 2030^1^. However, the current population-based one-size-fits-all clinical practice, where RT regimens are almost identical for all similar staged cancer patients, is sub-optimal due to the subtle physiological and genetic heterogeneity among individual patients that can result in diverse treatment responses and outcomes even when treated under identical protocols. Therefore, personalizing RT treatment according to a patient’s estimated treatment response is crucial for optimizing RT outcomes and ultimately increasing the rate of cancer survivorship. We hypothesize that a robust clinical decision support system (CDSS)^2^ that can quantitatively estimate long term treatment responses from pre and during treatment analyses and recommend proper prescription adjustments accordingly will be necessary to improve the standard of care in personalized RT.

**Figure 1:**
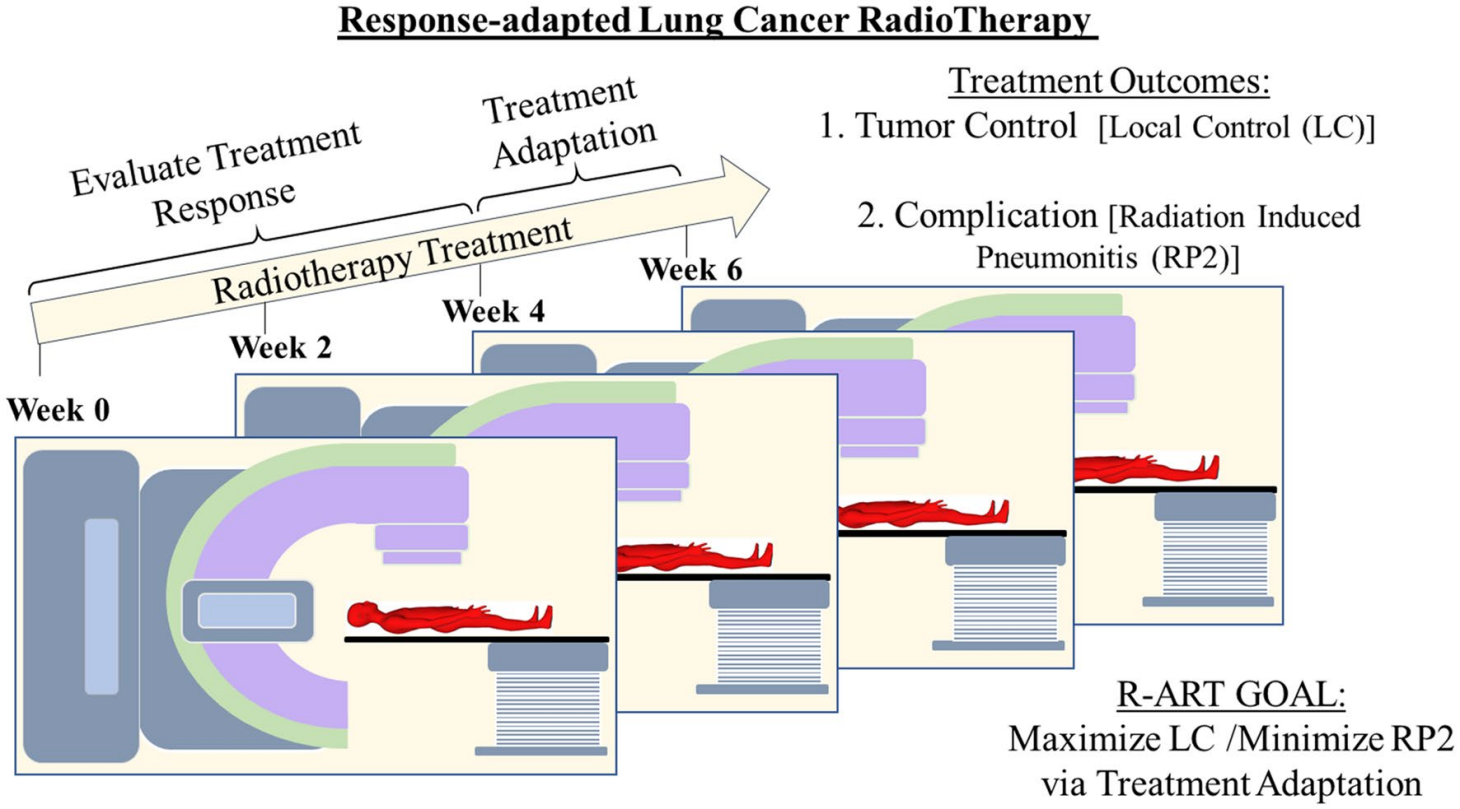
Schematics of response-adapted lung cancer radiotherapy. Response-adapted radiotherapy evaluates treatment response in the first two-thirds (week 1 to week 4) of the treatment period and then makes necessary adaptation in the last third (week 5 to week 6), with the goal of optimizing the treatment plan. For the case of lung cancer, optimization translates into maximizing tumor (local) control (LC) and minimizing radiation-induced pneumonitis of grade 2 or higher (RP2).

One proposed way of personalizing RT is shown in Fig. 1, which presents a schematic for knowledge-based response-adapted RT (KBR-ART)^3–5^. In a typical RT regimen for non-small cell lung cancer (NSCLC), a patient is given 60 Gray (Gy) of radiation in 30 fractions which takes a total of 6 weeks to administer^6^. The proposed KBR-ART will follow the initial treatment plan for the first two-thirds, or week 1 to week 4, of the regimen during which the patient’s treatment response is evaluated. Any necessary treatment adjustment (increase or decrease in dose per fraction) is carried out during the last one-third, or week 5 to week 6 of the regimen. The adjustment is intended to ensure an optimal treatment outcome, i.e., maximize tumor control and minimize radiation induced complication. Note that although we have chosen lung cancer for this study, the KBR-ART scheme is applicable to all types of cancer.

While the procedure for KBR-ART may be clear, obtaining the necessary information to create a standardized clinical protocol is difficult. The main challenge of KBR-ART is to quantify the relationship between radiation dose and treatment outcome. One way of measuring treatment response would be through direct (non-clinical; wide range of radiation dose) human experimentation, though such an approach would be unethical. Another challenge emerges from the complex biological processes that involves hundreds, if not thousands, of biological and clinical factors, which makes it an extremely difficult task to establish a quantitative relationship between radiation dose and treatment outcome, consequently complicating the task of making objective dose-adaptation decisions.

**Figure 2:**
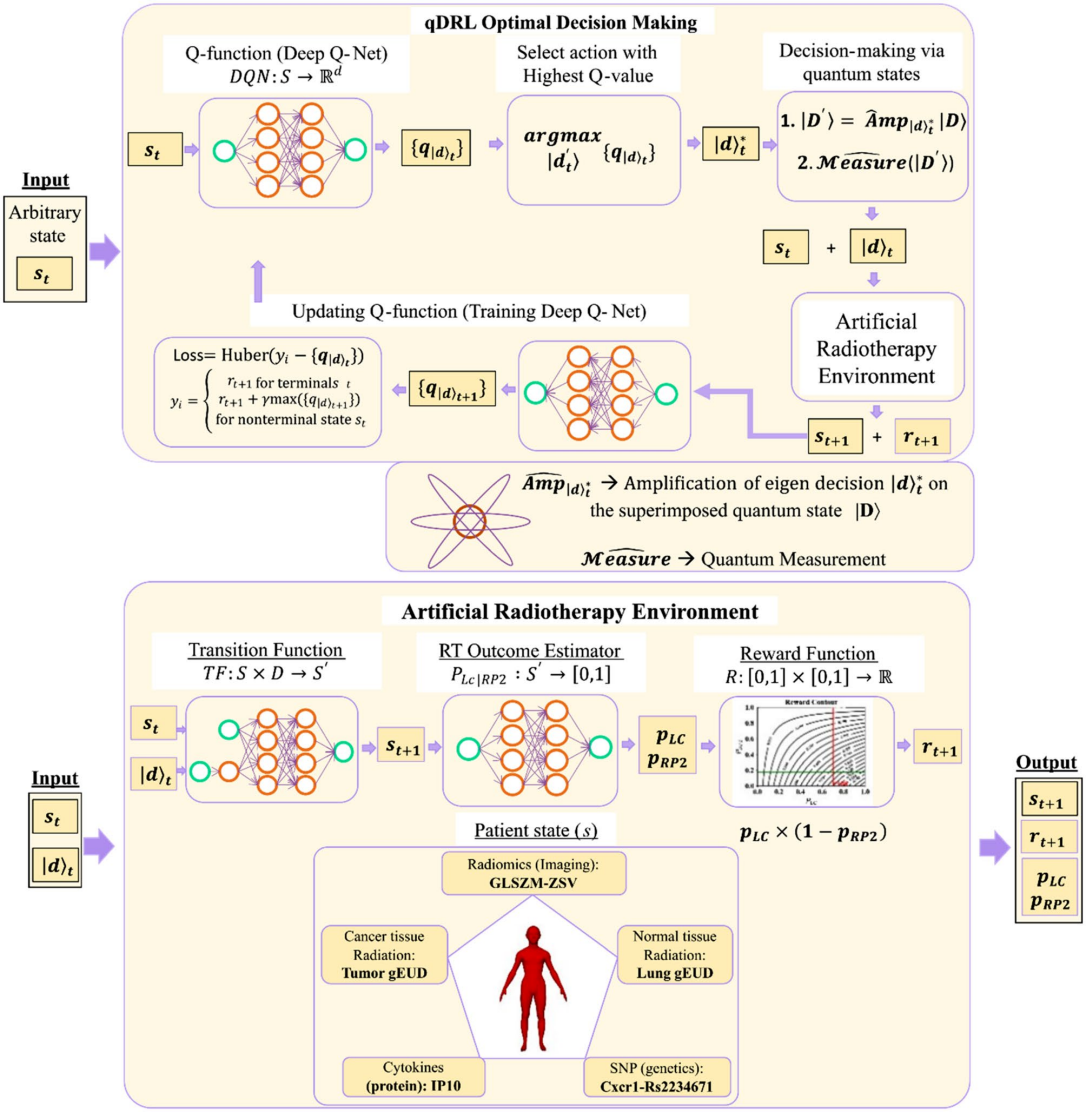
Quantum deep reinforcement learning algorithm for optimal decision making in knowledge-based adaptive radiotherapy. Schematic of a quantum deep reinforcement learning (qDRL) algorithm for optimal decision making in knowledge-based adaptive radiotherapy. qDRL employs deep q-net as a decision optimization algorithm and employs quantum state as the decision. Here, qDRL is a model-based algorithm that utilizes an artificial radiotherapy environment (ARTE) as the RL model. The qDRL artificially intelligent (AI) agent feeds in patient’s state $s_{t}$ in its memory (deep q-net) and obtains a set of q-values for a range of dose $(\left\{ {q_{{\left| d \right\rangle_{t} }} } \right\})$. The agent then selects the dose with the highest q-value and performs quantum amplification of that dose on a superimposed quantum dose decision state, $\left| D \right\rangle$. A quantum measurement is performed on the amplified state. The obtained dose measurement, $\left| d \right\rangle_{t}$, along with the state $s_{t}$ is fed into the ARTE. ARTE is composed of three functions in succession: (1) transition function, (2) RT outcome estimator, and (3) reward function, which predicts the patient’s next state $s_{t + 1}$, RT treatment outcome in terms of probability of local control, $p_{LC}$, and probability of radiation induced pneumonitis of grade 2 or higher, $p_{RP2}$, and reward value, $r_{t + 1} ,$ for the state-dose-decision pair. $s_{t + 1}$, and $r_{t + 1}$ are then used by the quantum agent to update its memory. This cycle is repeated until the agent finds a terminating state, after which a new cycle is initiated for a different patient. Five relevant biophysical features from radiomics, cancer and normal tissue radiation, cytokines, and genetics, were selected to represent the patient’s state based on our earlier work^13^.

Advanced machine learning methods such as deep learning, model-based reinforcement learning, and feature selection can alleviate the aforementioned challenges. Deep learning models are computational models that are made up of multi-layer neural networks that can combine data representation with the learning task in the same framework^7,8^. Deep learning techniques have considerably improved the field of computer vision, speech recognition, natural language processing, drug discovery and genomics, among other domains. Reinforcement learning (RL) is an area of machine learning concerned with teaching an artificially intelligent (AI) agent how to take optimal actions in a given environment to maximize a reward function^9^. Deep RL has also made a significant progress in creating AI systems that have even surpassed human-level intelligence in certain tasks such as Atari videogames^10^ and the Go board game^11^. Based on those advanced data driven techniques, we have designed a quantitative framework for KBR-ART using model-based reinforcement learning^12^ to model an artificial radiotherapy environment (ARTE) for dose adaptation optimization and using a Bayesian Network approach for relevant feature selection^13^. Our framework utilizes a hybrid of prior knowledge-based process-driven techniques and deep learning-based data-driven techniques in modeling an ARTE that can estimate RT treatment outcome for a given patient and radiation dose. The hybridization and application of prior knowledge provides a significant improvement to an earlier work by Tseng et al.^12^, which ensures that ARTE does not violate clinically observed facts which might not be easily apparent from the data alone. Incorporating prior knowledge also helped in mitigating overfitting pitfalls and reducing the model error; this allowed us to work with continuous numerical values, further improving the earlier work which only considered discrete values. Furthermore, we have defined the patients’ state in ARTE using the 5 important features selected from a multi-objective Bayesian network study^13^. A schematic of ARTE is presented in Fig. 2. For dose optimization, ARTE assigns a reward value for every pair of patient’s states and radiation dose, which is then accumulated by the deep reinforcement learning (DRL) algorithm in finding the optimal dose adaptation for that patient. However, even with the improvements to ARTE, the earlier approach alone is insufficient to model the intrinsic ‘noise’ in patient’s data generated by the uncertainty in physician’s decision-making process.

In this work, we have introduced a novel modeling approach for clinical application, where we model the clinical decisions with quantum states to represent the indeterminism in the clinical decision-making process during RT treatment due to the unavailability of complete information on RT treatment course and outcomes. Decisions made under uncertainty are typically influenced by personal preference^14^, which are subject to change with varying information in a situation, and several experiments have demonstrated preference reversal in human decision-making^15,16^. Considerable efforts have been made to describe anomalies in human decision-making, such as preference reversal, based on the laws of classical probability theory, in terms of subjective utility and subjective probability^17,18^; however, they have not been successfully generalized, and several other experiments have shown violation of these principles^19^. These inadequacies of classical decision-making theory can be overcome by quantum probability theory-based models of human decision-making processes^16,20–22^. Because quantum states are noncommutative, quantum probabilities are intrinsically asymmetric, providing a consistent mathematical framework to overcome the shortcomings of classical probability that primarily arises from its symmetrical property.

We, however, directly modeled human decision as quantum states and decision-selection as quantum interaction, bypassing the utility-based quantum theoretic approach. Our approach is similar to the value-based quantum reinforcement learning (QRL) framework by Li et al.^23–25^. Li et al. in their study^25^ demonstrated a QRL framework to be a better alternative for mimicking human decision-making, in which 2 QRL and 12 classical RL models were compared in modeling human decision-making among healthy and cigarette-smoking subjects while performing the Iowa Gambling Task. The authors argued that quantum-like features exist in human decision-making, in which making a choice can influence the subjective values of the alternatives; since quantum states can exist in a superimposed state, (i.e., a state representing all the alternatives at once) any operation, for instance selecting a choice, will influence the value of all other alternatives.

Our framework, though similar in concept with Li’s QRL, varies in its implementation. We have employed deep q-net^26^ technology for optimizing decision prediction and to provide a more complete representation of a patient’s state due to its ability to function with nonlinear complex systems over a continuous space. Additionally, we have designed a novel quantum controller circuit that allows our framework to run on a quantum processor for modeling decisions with realistic quantum states and decision-selection with real quantum interaction. By combining the quantum computation and deep q learning, we have developed a novel quantum deep reinforcement learning (qDRL) framework for modeling optimal decision-making processes in KBR-ART (Fig. 2).

In this work, we demonstrate the first application of a qDRL framework to real-world clinical data for decision-making, particularly in the case of NSCLC. For comparison, we trained and validated three frameworks: DRL, qDRL trained on a Qiskit quantum computing simulator^27^, and qDRL trained on an IBM quantum (IBMQ) 16 Melbourne 15-qubit processor^28^. We trained our framework on 67 stage III NSCLC patient datasets from a single institution^13^ and validated our framework on an independent multi-institutional cohort of 174 NSCLC patients treated under the Radiation Therapy Oncology Group- (RTOG-) 0617 protocol^29^.

This paper is organized as follows. In the Results section, we present the AI recommendations generated from three RL frameworks and compare the results with one another and the clinical decisions. Since our framework is not a supervised learning algorithm, a direct performance comparison is not possible; instead, we developed comparison metrics based on the radiation dose to treatment outcome relationship. The Discussion section presents the analysis of the results and describes the limitations and the potential of our framework. The Methods section describes the details of our framework including the mathematical details of qDRL, the design of a novel quantum amplification method called a Controller Circuit, the description of the utilized patient features, and details of the various components of the proposed ARTE.

## Results

**Figure 3:**
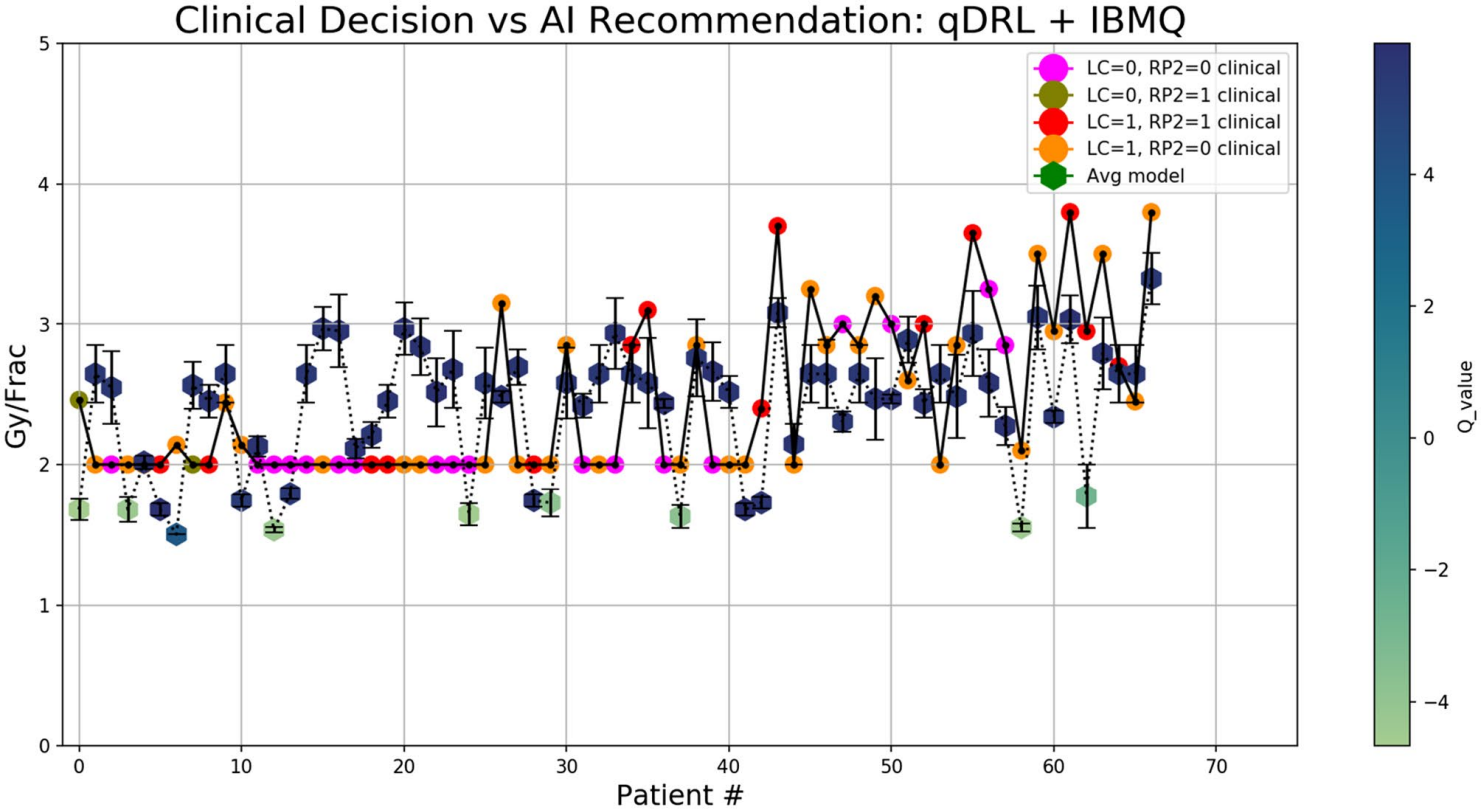
Clinical decision support system dose adaptation recommendation. Comparison between retrospective clinical decision and AI recommendations obtained from the quantum deep reinforcement learning (qDRL) model trained in the IBMQ quantum processor. Each datapoint corresponds to the dose decision (dose per fraction) for the last one-third treatment period for the training dataset. The clinical data points are color-coded according to the four possible binary clinical outcomes: LC = 0 (no local control) and RP2 = 0 (no radiation induced pneumonitis of grade 2 or higher), LC = 0 and RP2 = 1, LC = 1 and RP2 = 1, and LC = 1 and RP2 = 0. Here LC = 1 and RP2 = 0 is the clinical desirable outcome. The AI recommendation is color-coded according to the q-value (expected return of recommending a dose), which can be loosely interpreted as the AI’s confidence in its recommendation. Here, the AI recommendations are averages of 5 identically trained models with standard error of mean as uncertainty. For clarity, this figure is rotated and presented in landscape format in the Supplementary Material.

Optimal AI recommendations (dose per fraction for the last 1/3 of treatment) obtained from qDRL models trained in the IBMQ quantum processor are shown in Fig. 3. AI recommendations from other models are presented in the Supplementary Material. We repeated every experiment 5 times for rigorous statistical reporting of the average recommendations and standard error of mean as uncertainty as shown in Fig. 3. For visual comparison, we added the retrospective clinical dose decision for each patient, color-coded with the respective clinical outcomes. Altogether the binary outcomes can be classified into 4 classes, among which the class with successful local tumor control (LC = 1) and no occurrence of radiation induced pneumonitis (RP2 = 0) is the clinically desirable outcome.

For additional information on the AI decision-making, we have color-coded AI recommendations with their corresponding q-value (quality value) obtained from the deep q-net. Deep q-net serves as the memory of the AI agent, which assigns a q-value for every possible pair of the patient’s state and dose decision. Mathematically, the q-value is the expected return (time-average) of recommending a series of dose to a patient until the AI agent either achieves the desirable treatment outcome estimates or exceeds a user-defined terminating steps (in our case 10 steps). At every step of dose decision-making, the AI agent gets a reward based on the treatment outcome estimates, which is then aggregated into q values throughout the training process. We assigned positive rewards for successfully attaining the desirable outcomes and negative rewards for failing to do so. Since an agent always selects the greedy decision (decisions with the maximum q value), the q-value can be loosely translated as the AI’s confidence in a recommended dose. A positive q-value corresponds to the decision that most likely results in a desirable outcome, and while a negative q-value still corresponds to the best decision for a particular case, it is less likely to result in a desirable outcome. Note that the absolute magnitude of q value will change with different reward scheme and training parameters.

Because our framework is not supervised learning, we devised the following 2 complementary metrics for evaluating model performance.

1. *Similarity score* (root mean square error [RMSE] between retrospective clinical decisions and the AI recommendation): Similarity score provides a direct metric to compare the closeness of the AI recommendation to the clinical practice. Although the goal of our modeling is not to replicate the retrospective clinical decision, because the KBR-ART paradigm is based on the premise that the current practice is suboptimal, the RMSE metric can still provide some level of information regarding the quality of the model since the current practice is based on clinically validated measures. This means that a 0 Gray/fraction (Gy/frac) RMSE similarity score is undesirable, however a low RMSE generally indicates a good recommendation.

2. *Self-evaluation scheme* (evaluation based on the radiation dose to outcome relationship). This scheme is also based on retrospective clinical decisions but factors in the clinical outcomes^12^. The scheme is summarized in Table 1, which assumes that higher radiation dose simultaneously increases the probability of controlling tumor as well as the risk of complications. This assumption is based on the observation that radiation kills both cancer cells and healthy cells. For instance, if the clinical outcome for a patient was no local control (LC = 0) and no occurrence of radiation induced pneumonitis (RP2 = 0), then an AI dose recommendation higher than the clinical decision can be considered as a good recommendation (i.e., the patient would have benefited from dose escalation at week 4 of the treatment period) and vice-versa. Out of the four possible permutations, LC = 1 and RP2 = 0 is the only clinically optimal outcomes. Since its impossible to evaluate recommendations for the desirable outcomes, we have used ‘unsure’ and ‘good’ tags for the AI recommendation. When an AI recommendation falls outside 0.5 Gy/frac of the clinical dose decision, we tag the recommendation as ‘unsure’. This uncertainty is due to the lack of knowledge about individual patients’ sensitivity to dose adaptation. However, when an AI recommended dose falls inside the 0.5 Gy/frac margin of the clinical decision, we can tag the dose as a good recommendation with higher confidence. Among the clinically undesirable outcomes, patients with LC = 0 and RP2 = 0 could have received a higher dose to increase the chance of LC while patients with LC = 1 and RP2 = 1 could have received a lower dose to decrease the chance of RP2. However, for LC = 0 and RP2 = 1, the outcome of increasing or decreasing the dose is unclear. We took into consideration the fact that, clinically, the amount of dose administered is limited by the likelihood of causing RP2—the side-effect on any treatment should be minimized. So, when AI recommends a dose that is less than the clinical dose decision, we tag it as a ‘good’ recommendation. Additionally, for assurance, we added a cushion of 0.1 Gy/frac and defined ‘good’ as the AI recommendation that is less than clinical decision minus 0.1 Gy/frac.

**Table 1:** Self-evaluation scheme.

LC	RP2	Dose difference ($\Delta$)^a^	Remark
0	0	$\Delta \le 0$	Bad
0	0	$\Delta > 0$	Good
0	1	$\Delta < - 0.1$	Good
0	1	$\Delta \ge - 0.1$	Bad
1	0	$\left| \Delta \right| \le 0.5$	Good
1	0	$\left| \Delta \right| > 0.5$	Unsure
1	1	$\Delta < 0$	Good
1	1	$\Delta \ge 0$	Bad

Evaluation scheme for AI recommendation based on the radiation dose to outcome relationship—the probability of both LC and RP2 increases with increase in radiation dose. Here LC and RP2 are retrospective treatment outcome and LC = 1 and RP2 = 0 are the clinically desired outcome. For a patient with known treatment outcome, we can evaluate an AI recommendation by comparing it with the retrospective clinical dose decision. For instance, for a patient with LC = 0 and RP2 = 0, we can say that the patient should have been given a higher dose.

^a^$\Delta$ = AI recommendation – Clinical decision.

0: no event, 1: event.

*LC* local control, *RP2* radiation-induced pneumonitis of grade 2 or higher.

**Figure 4:**
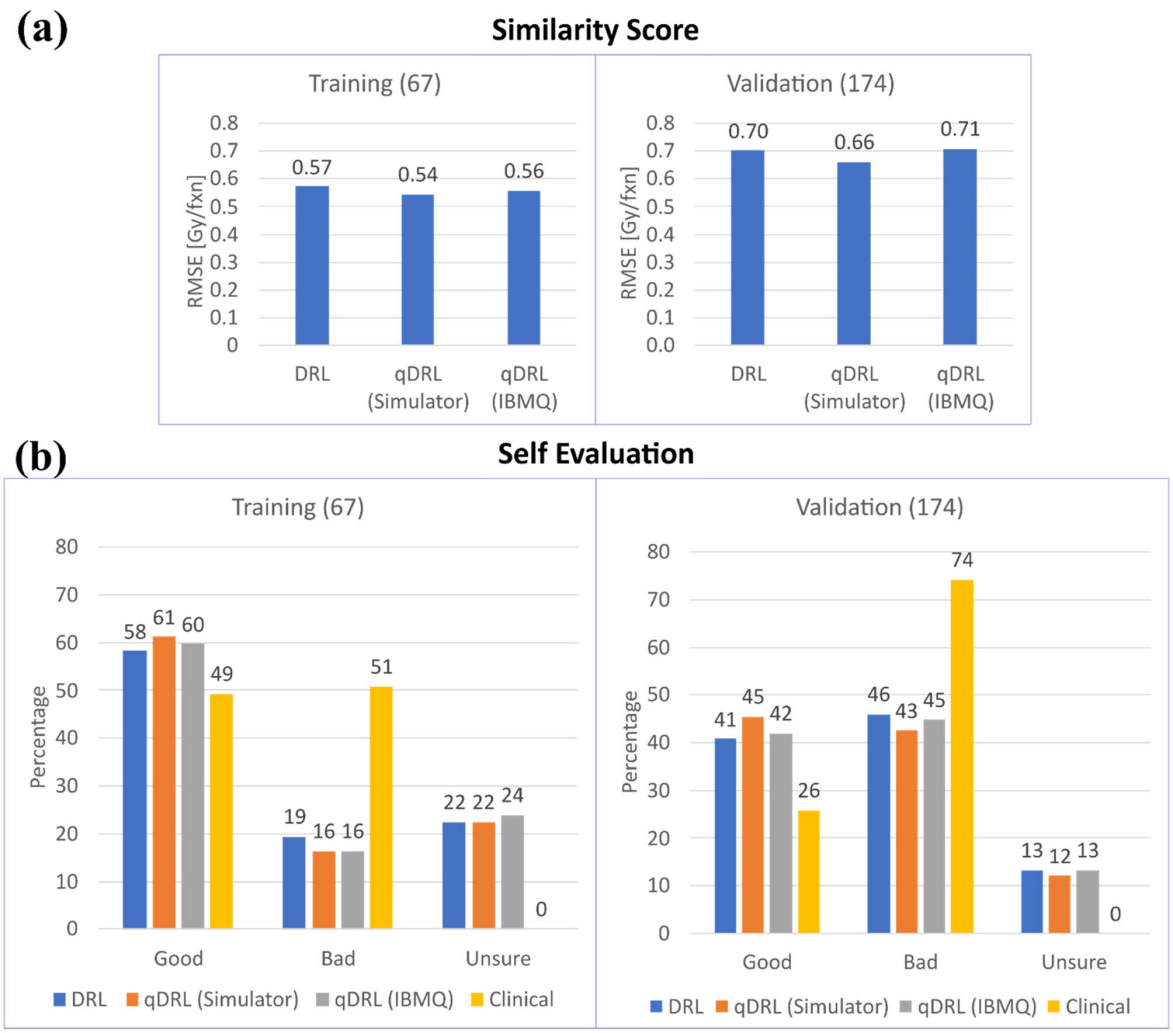
Training and validation results. (**a**) similarity scores and (**b**) self-evaluation results for the training and validation data sets. A total of 5 numerical experiments were carried out for each of the models: Deep reinforcement learning (DRL), quantum DRL (qDRL) trained in simulator, and qDRL trained in IBMQ quantum processor. In particular, we used Double DQN algorithm for DRL. All the results correspond to the average model. The results of the Self-Evaluation I scheme are converted into the percentage of the total patients. The similarity score is the root mean square error (RMSE) value between the retrospective clinical dose decision and the AI recommendation while the self-evaluation value is calculated based on the clinical outcome and the clinically established relationship between radiation dose and RT outcome.

Analysis of model performance based on the above two metrics is presented as bar charts in Fig. 4. Similarity scores for DRL, qDRL trained in Qiskit Simulator (qDRL + simulator), and qDRL trained in IBMQ quantum processor (qDRL + IBMQ) for the training dataset were 0.57 Gy/frac, 0.54 Gy/frac, and 0.55 Gy/frac, respectively, and for the validation dataset were 0.70 Gy/frac, 0.66 Gy/frac, and 0.71 Gy/frac, respectively. The similarity scores were calculated between the averaged recommendations and the clinical decisions. The results from the self-evaluation scheme were converted into percentage of the total number of patients. The self-evaluation results for the training datasets are as follows: the percentages of good recommendations for DRL, qDRL + simulator, qDRL + IBMQ, and clinical dose were 58%, 61%, 60%, and 49%, respectively; the percentages of bad recommendations were 19%, 16%, 16%, and 51%, and the percentages of unsure recommendations were 22%, 22%, 24%, and 0%. Similarly, the self-evaluation results for the validation datasets are as follows: the percentages of good recommendations for DRL, qDRL + simulator, qDRL + IBMQ and clinical dose were 41%, 45%, 42%, and 26%, respectively; the percentages of bad cases were 46%, 43%, 45%, and 74%, and the percentages of unsure cases were 13%, 12%, 13%, and 0%. Note that the analysis of clinical doses is based on the clinical outcomes.

## Discussions

Analysis of our result indicates that our framework can potentially improve clinical RT treatment outcome by at least about 10%. This metric corresponds to the minimum difference between the percentage of good recommendations and clinical decision as shown in Fig. 4, for the combined training and validation results. Precisely, it’s the difference between DRL and the clinical decision. We recognize that the self-evaluation scheme alone provides partial information on the performance, thus we base our conclusion by considering both the self-evaluation scheme and similarity score; the similarity score shows that our framework is close to clinical decisions with a RMSE value of at most 0.71 Gy/frac. With a smaller similarity score and better performance under the self-evaluation scheme, we have shown that our framework can recommend doses close to medical experts with a higher chance of successful treatment of NSCLC patients. However, we would like to acknowledge that our analysis depends on having an accurate representation of the RT environment model and the accuracy of the outcome estimator. Modeling of a multimodal treatment environment including immunotherapy and surgical and medical oncology will be necessary for a more comprehensive assessment of our framework.

Our analysis showed that qDRL + simulator models are slightly different than the qDRL + IBMQ. The reason for this difference is twofold: (1) the simulator lacks any machine error including the quantum decoherence error (i.e., inability of maintaining a coherent quantum phase), (2) the decision selection mechanism of qDRL + simulator is different from qDRL + IBMQ. The former reason results in higher noise but could be more representative of the human decision-making process. The latter reason comes from the physical necessity to design a quantum circuit of length shorter than the quantum coherence length of a quantum processor. Any quantum algorithm (strictly speaking its equivalent quantum circuit) that is longer than the system’s coherence length will result in error. We designed a novel quantum controller circuit that was much shorter, thus easier to design and use, than the Grover amplification process (the decision selection mechanism of qDRL + simulator derived form QRL). Note that the quantum controller circuit is by no means a replacement of the Grover’s amplification process as a search algorithm; it is, however, a pragmatic and scalable alternative for the decision selection mechanism.

Although our analysis showed only a small improvement in performance between DRL and the qDRL algorithm, our hybrid quantum–classical machine learning algorithm possesses both conceptual and computational advantages. We found qDRL models exhibited slightly smaller similarity score than the DRL models, i.e., about 0.03 Gy/frac difference for training dataset and about 0.04 Gy/frac difference for validation dataset (best case). Similarly, in terms of self-evaluation scheme, qDRL models made more good recommendations and less bad recommendations, i.e., about 3% difference for both good and bad recommendations for both training and validation dataset (best case). While it is difficult to favor the qDRL algorithm over the DRL algorithm solely based on the performance metrics, the qDRL algorithm has the following two advantages: (1) modeling of human decision with quantum state has a sound theoretical background and (2) a hybrid quantum–classical machine learning algorithm can harness quantum properties such as superposition, parallelism, and entanglement. In this work we designed our framework with value based DRL methods and, as a next iteration, plan to explore and incorporate advanced policy based DRL methods to our qDRL framework for additional improvement.

Our approach of combining quantum computation, deep learning techniques, and statistical ensemble provides the necessary robustness for a clinically viable CDSS. Modeling of human-decision as a quantum state is not only theoretically consistent but also takes into consideration the intrinsic indeterminism of the human decision-making process. Deep learning techniques use redundancy of a large number of nodes to handle (desensitize) data noise. Averaging over the ensemble of multiple trained models makes our approach less sensitive to stochastic training methods used in deep learning training. For clinical application, further improvement is necessary, which can be achieved by using a larger training dataset, further infusing expert knowledge in modeling of ARTE, more independent validation and testing, and the development of a graphical user interface. Additionally, testing our framework in a prospective study will be necessary for future clinical implementation^30^.

Aside from data-related limiting factors, our framework will have to overcome several other limitations and consider concurrent complimentary technologies such as modern radiotherapy delivery techniques for future clinical application. Considering a patient-specific alpha-to-beta ratio would capture the heterogeneity among patients and boost the efficiency of the current framework. Combining the volatility of radiomics features for PET images into the uncertainty analysis would provide a more accurate representation of the predictability of the framework. Similarly, an extensive sensitivity analysis of the features will be necessary for better representation of the response prediction, range of variability, and the uncertainty estimates of our framework. In terms of application, our framework should be used in a complementary fashion and consistent with other clinical tools such as the RT treatment planner, plan optimizer, and image guidance system^29^ for clinical implementation.

In summary, a framework for a robust CDSS based on quantum computing and deep learning methods was designed and tested. The key feature of the framework was modeling the clinical decisions as quantum states. The novelty of our approach lies in combining quantum computation with deep learning to gain the best of both fields. A novel quantum circuit was designed and implemented in IBMQ quantum processor to utilize real quantum states and quantum interaction. Two complementary schemes were designed to analyze the performance of our weakly supervised framework. Based on our analysis, our framework showed potential of improving RT efficacy. Finally, our framework provides a clinically viable quantitative approach to decision-making in KBR-ART.

## Methods

### Quantum deep reinforcement learning

Quantum deep reinforcement learning is a novel action value-based decision-making framework derived from QRL^23^ and deep q-learning^10^ framework. Like conventional RL^9,31^, our qDRL based CDSS framework is comprised of 5 main elements: clinical AI agent, ARTE, radiation dose decision-making policy, reward, and q-value function. Here, the AI agent is a clinical decision-maker that learns to make dose decisions for achieving clinically desirable outcomes within the ARTE. The learning takes place by the agent-environment interaction, which can be sequentially ordered as: the AI decides on a dose and executes it, and in response, a patient (part of the ARTE) transits from one state to the next. Each transition provides the AI with feedback for its decision in terms of RT outcome and associated reward value. The goal of RL is for the AI to learn a decision-making policy that maximizes the reward in the long run, defined in terms of a specified q-value function that assigns a value to every state-dose-decision pair obtained from the accumulation of rewards over time (returns).

Assuming Markov’s property (i.e., an environment’s response at time $t + 1$ depends only on the state and dose-decision at time $t$), the qDRL task can be mathematically described as a 5-tuple $(S, \left| D \right\rangle , TF, P, R)$, where $S$ is a finite set of patient’s states, $\left| D \right\rangle$ is a superimposed quantum state representing the finite set of eigen-dose decision, $TF:S \times D \to S^{\prime }$ is the transition function that maps patient’s state $s_{t}$ and eigen-dose $\left| d \right\rangle_{t}$ to the next state $s_{t + 1}$, $P_{LC|RP2} :S^{\prime } \to \left[ {0,1} \right]$ is the RT outcome estimator that assigns probability values $p_{LC}$ and $p_{RP2}$ to the state $s_{t + 1}$, and $R:\left[ {0,1} \right] \times \left[ {0,1} \right] \to {\mathbb{R}}$ is the reward function that assigns a reward $r_{t + 1}$ to the state-decision pair $\left( {s_{t} ,\left| d \right\rangle_{t} } \right)$ based on the outcome probability estimates.

Eigen-dose $\left| d \right\rangle$ is a physically performable decision that is selected via quantum methods from the superimposed quantum state $\left| D \right\rangle$ which simultaneously represents all possible eigen-doses at once. In simple words, $\left| D \right\rangle$ is the collection of all possible dose options and $\left| d \right\rangle$ is one of those options which is selected after a decision is made. Selecting dose decision $\left| d \right\rangle$ is carried out in two steps: (1) amplifying the optimal eigen-dose $\left| d \right\rangle^{*}$ from the superimposed state $\left| D \right\rangle$ (i.e., $\left| D \right\rangle^{\prime } = \widehat{Amp}_{{\left| d \right\rangle^{*} }} \left| D \right\rangle$) and (2) measuring the amplified state (i.e., $\left| d \right\rangle = \widehat{Measure}(\left| {D^{\prime } } \right\rangle )$.

The optimal eigen-dose $\left| d \right\rangle^{*}$ is obtained from deep Q-net, which is the AI’s memory. Deep Q-net, $DQN:S \to {\mathbb{R}}^{d}$, is a neural network that takes patient’s state as input and then outputs q-value for each eigen-dose ($\left\{ {q_{\left| d \right\rangle } } \right\}$). The optimal dose is then selected following greedy policy where the dose with the maximum q-value is selected (i.e., $$\left| d \right\rangle^{*} = \begin{array}{*{20}c} {argmax} \\ {\left| {d^{\prime } } \right\rangle } \\ \end{array} \{ q_{\left| d \right\rangle } \}$$). We have applied a double Q-learning ^32^ algorithm in training the deep Q-net. The schematic of a training cycle is presented in Fig. 2 and additional technical details are presented in the Supplementary Material.

### Quantum amplification method and controller circuit

We initially employed Grover’s amplification procedure^33,34^ for the decision selection mechanism. While Grover’s procedure works on a quantum simulator, it fails to correctly work in a quantum computer. The quantum circuit depth of Grover’s procedure (for 4 or higher qubits) is much greater than the coherence length of the current quantum processor^35^. Whenever the quantum circuit length exceeds the coherence length, quantum state becomes significantly affected by the system noise and loses vital information. Therefore, we designed a quantum controller circuit that is shorter than the coherence length and is suitable for the task of decision selection. The merit of our design is its fixed length; since its length is fixed for any number of qubits, it is suitable for higher qubit systems, as much as permitted by the circuit width. Technical details regarding its implementation in quantum processor is presented in the Supplementary Materials.

**Figure 5:**
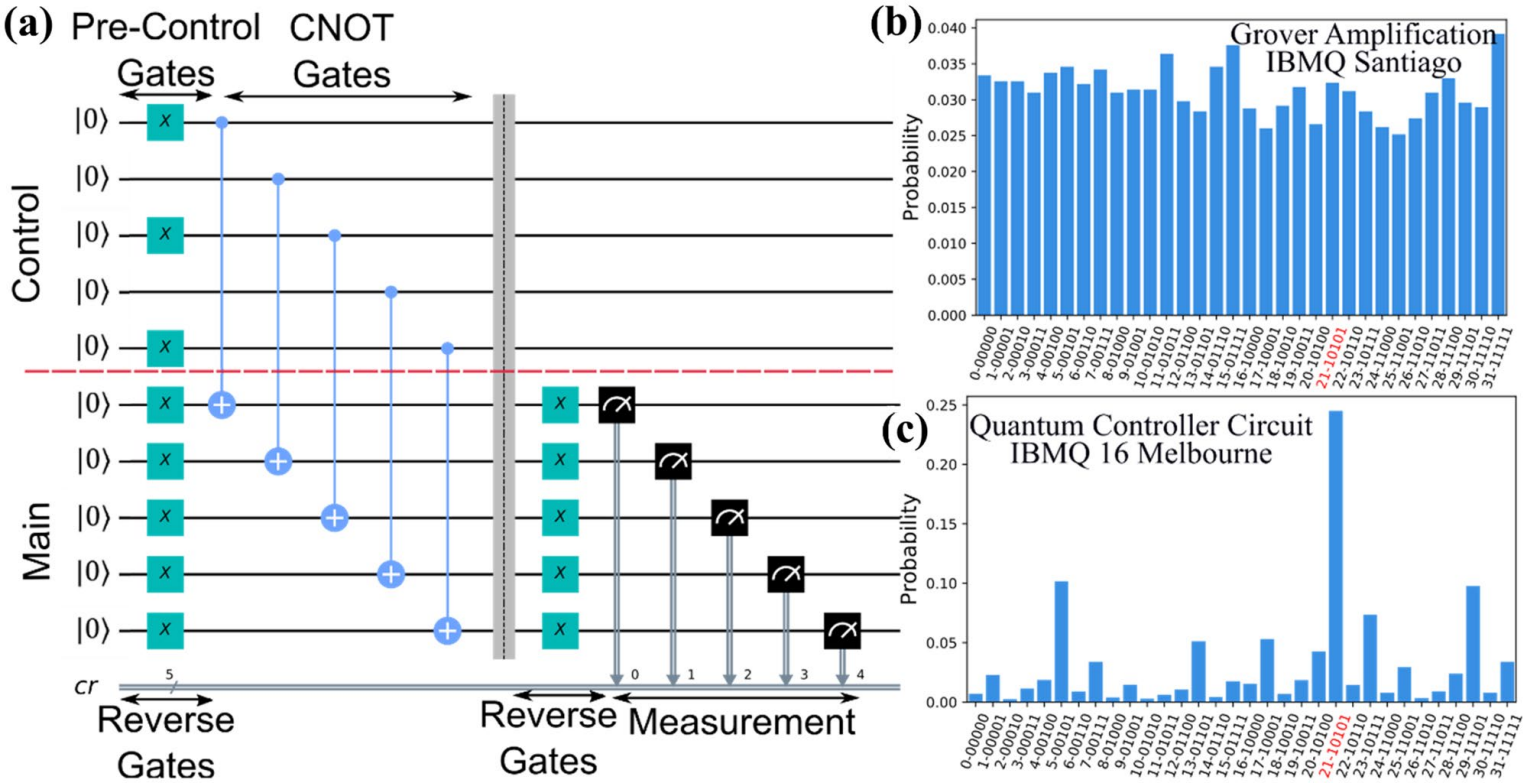
Quantum controller circuit for a 5 qubit (32 bit) system. (**a**) Quantum controller circuit for the selection of the state $\left| {10101} \right\rangle$. The probability distribution corresponding to (**b**) failed Grover’s amplification procedure for one iteration run in the 5-qubit IBMQ Santiago quantum processor and (**c**) successful quantum controller selection run in the 15-qubit IBMQ Melbourne quantum processor.

An example of a controller circuit is given in Fig. 5. Controller circuits use twice the number of qubits (n), which can be divided into control and main. Optimal eigen-states obtained from deep Q-net are created in the control by selecting the appropriate pre-control gates. Then the control is entangled with the qubits from the main via controlled NOT (CNOT) gates. CNOT gates are connected between a control qubit from the control and a target qubit from the main. CNOT gates flip the target qubit from $\left| 1 \right\rangle$ to $\left| 0 \right\rangle$ only when the control is in $\left| 1 \right\rangle$ state and does not perform any operation otherwise. Because all the main qubits are prepared in $\left| 0 \right\rangle$ state, we introduced the reverse gates (n X-gates in parallel) to flip them to $\left| 1 \right\rangle$. X-gates flip $\left| 0 \right\rangle$ to $\left| 1 \right\rangle$, and vice-versa. The CNOT flips all the qubits whose controls are in $\left| 1 \right\rangle$ state, creating a state that is element-wise opposite to the marked state. Finally, another set of reverse gates is applied to the main before making a measurement.

Another advantage of the controller circuit is controlled uncertainty level. The controller circuit has additional degrees of freedom that can control the level of uncertainty that might be needed to model a highly dubious clinical situation. By replacing the CNOT gate by a more general $CU3\left( {\theta ,\phi ,\lambda } \right)$ gate, we can control the level of additional stochasticity with the rotation angles $\theta$, $\phi$, and $\lambda$, which corresponds to the angles in the Bloch sphere. The angles can either be fixed or, for additional control, changed with training episode.

### Patient characteristics

The patient’s state in the ARTE is defined by 5 biological features: cytokine (IP10), PET imaging feature (GLSZM-ZSV), radiation doses (Tumor gEUD and lung gEUD), and genetics (cxcr1- Rs2234671). Their descriptions are presented in Table 2. These 5 variables were selected from a multi-objective Bayesian Network study^13^, which considered over 297 various biological features and found the best features for predicting the joint LC and RP2 RT outcomes.

**Table 2:** Description of the patients’ features.

Patient variable	Biological/clinical characteristics	References
IP10 (Cytokine/Signaling molecule)	IP10 (Interferon gamma-induced protein 10) is secreted in response to IFN-γ by various cells including monocytes, endothelial and fibroblasts. (i) Acts as chemoattractant for monocytes/macrophages, T cells, NK (natural killer) cells, and dendritic cells. (ii) Promotes T cell adhesion to endothelial cells. (iii) Antitumor activity (iv) Inhibition of bone marrow colony formation (v) Angiogenesis	^36–38^
GLSZM-ZSV (Tumor PET Imaging features/Radiomics)	Radiomics features: the zone-size variance (ZSV) feature of a gray-level size zone matrix (GLSZM) is defined as $\frac{1}{{N_{g} \times L_{z} }}\mathop \sum \limits_{{N_{g} }}^{i = 1} \mathop \sum \limits_{{L_{z} }}^{j = 1} \left( {jp\left( {i,j} \right) - \mu_{j} } \right)^{2}$, refer to Appendix A5 of Ref.^34^ for the Notations	^39^
Tumor gEUD (tumor radiation)	Generalized equivalent uniform dose (gEUD) of tumor converted from EQD2 (Equivalent Dose at standard 2 Gy per fraction) dose distribution: $gEUD = \left( {\mathop \sum \limits_{i} \nu_{i} {\text{eqd}}_{2}^{a} } \right)^{\frac{1}{a}}$ and ${\text{eqd}}2 = N_{frac} \times d \times \frac{d + \alpha /\beta }{{2 + \alpha /\beta }}$ *where* $\alpha /\beta$ = 10 Gy, is the radiation fractionation sensitivity of cell, a = − 10 is an organ specific parameter, and $\nu$ is the fractional organ volume obtained from the 3D dose distribution	^40^
Lung gEUD (lung radiation)	Generalized equivalent uniform dose (gEUD) of lung converted from EQD2 (Equivalent Dose at standard 2 Gy per fraction) dose distribution: $gEUD = \left( {\mathop \sum \limits_{i} \nu_{i} {\text{eqd}}_{2}^{a} } \right)^{\frac{1}{a}}$ and ${\text{eqd}}2 = N_{frac} \times d \times \frac{d + \alpha /\beta }{{2 + \alpha /\beta }}$ *where* $\alpha /\beta$ = 4 Gy, and a = 1	^40^
Cxcr1-Rs2234671 (genetics)	A single nucleotide polymorphism (SNP) in the gene cxcr1, also known as Interleukin 8 receptor alpha (IL8RA), related to radiation induced toxicity in non-small cell lung cancer. SNP is a substitution of single nucleotide that occurs at a specific position in the genome via mutation	^41^

Description of the five features used to define patients’ state in the artificial radiation therapy environment of our framework.

The training data analyzed in this study are obtained from the University of Michigan study UMCC 2007.123 (NCI clinical trial NCT01190527) and the validation data analyzed in this study are obtained from the RTOG-0617 study (NCI clinical trial NCT00533949). Both trials were conducted in accordance with relevant guidelines and regulations and informed consent was obtained from all subjects and/or legal guardians. Details on training and validation datasets, and necessary model imputation carried out to accommodate the differences in the datasets are presented in the Supplementary Materials.

### Transition function

Deep Neural Networks (DNN) were applied as transition functions for IP10 and GLSZM-ZSV features. They were trained with a longitudinal (time-series) dataset, with the pre-irradiation patient state and corresponding radiation dose as input features and post-irradiation state as output. For lung and tumor gEUD, we utilized prior knowledge and applied a monotonic relationship for the transition function since we know that gEUD should increase with increasing radiation dose. We assumed that the change in gEUD is proportional to the dose fractionation and tissue radiosensitivity,

1 $$\frac{{g\left( {t_{n} } \right) - g\left( {t_{n - 1} } \right)}}{{t_{n} - t_{n - 1} }} \propto d_{n} \left( {1 + \frac{{d_{n} }}{{\frac{\alpha }{\beta }}}} \right).$$

Here $g\left( {t_{n} } \right)$ is the gEUD at time point $t_{n}$, $d_{n}$ is the radiation dose fractionation given during the n^th^ time period, and $\alpha /\beta$ ratio is the radiosensitivity parameter which differs between tissue type. Note that we first applied constrained training^42^ to maintain monotonicity with DNN model, however the gEUD over time trend was flatter than anticipated, thus we opted for a process-driven approach in the final implementation. The technical details on the NNs and its training are presented in the Supplementary Material.

### RT outcome estimator

DNN classifiers were applied as the RT outcome estimator for LC and RP2 treatment outcomes. They were trained with post irradiation patient states as input and binary LC and RP2 outcomes as its labels.

RT outcome estimator must also satisfy a monotone condition between increasing radiation dose and increasing probability of local control as well as probability of radiation induced pneumonitis. To maintain this monotonic relationship, we used a generic logistic function,

2 $$p_{LC|RP2} = \frac{1}{{1 + \exp \left( {\frac{{g\left( {t_{6} } \right) - \mu }}{T}} \right)}},$$

where $g\left( {t_{6} } \right)$ is the gEUD at week 6, and $\mu$ and $T$ are two patient-specific parameters that are learned from training the DNN. Here, $\mu$ and $T$ are the outputs of two neural networks that are fed into the logistic function and tuned one after the other, leaving the other fixed. The training details are presented in the Supplementary Materials.

### Reward function

**Figure 6:**
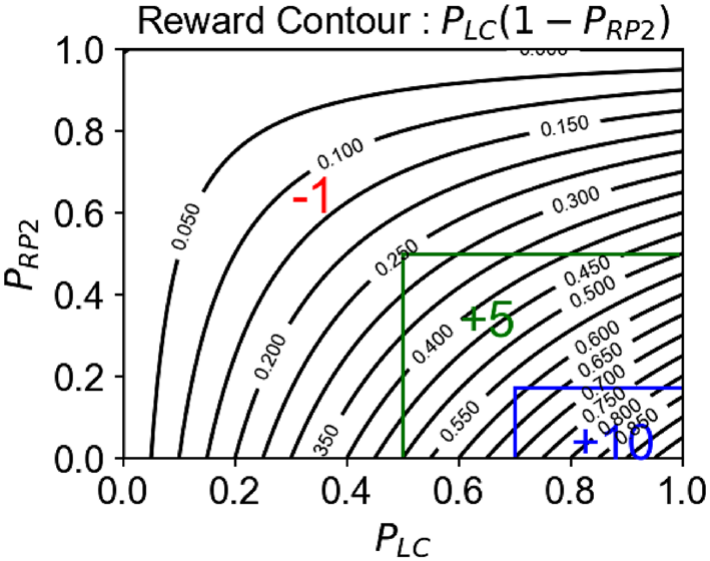
Reward function for reinforcement learning. Contour plot of reward function as a function of the probability of local control (PLC) and radiation induced pneumonitis of grade 2 or higher (PRP2). Area enclosed by the blue line corresponds to the clinically desirable outcome, i.e., $$P_{LC} > 70{\%}$$ and ${P_{RP2}} <17.2{\%}$ . Similarly, the area enclosed by the green lines corresponds to the computationally desirable outcome, i.e., $$P_{LC} > 50{\%}$$  and ${P_{RP2}} <50{\%}$. Along with $$P_{LC} \times (1-P_{RP2})$$ the AI agent receives + 10 reward for achieving clinically desirable outcome, + 5 for achieving computationally desirable outcome, and -1 when unable to achieve a desirable outcome.

The task of the agent is to determine the optimal dose that maximizes $p_{LC}$ while minimizing $p_{RP2}$. Accordingly, we built a reward function on the base function $P^{ + } = P_{LC} \left( {1 - P_{RP2} } \right)$ as shown in Fig. 6. The algebraic form is as follows,

3 $$R = \left\{ {\begin{array}{*{20}l} {P^{ + } + 10 } \hfill & { {\text{if}} 70\% < p_{Lc} < 100\% \;{\text{and}}\; 0\% < p_{RP2} < 17.2\% } \hfill \\ {P^{ + } + 5} \hfill & {{\text{if}} 50\% < p_{Lc} < 70\% \;{\text{and}}\; 17.2\% < p_{RP2} < 50\% } \hfill \\ {P^{ + } - 1} \hfill & {{\text{if}} 0\% < p_{Lc} < 50\% \;{\text{and}}\; 50 < p_{RP2} < 100\% } \hfill \\ \end{array} } \right.$$

Here the AI agent receives additional 10 points for achieving clinically desirable outcome (i.e., $p_{LC} > 70\% \quad {\text{and}} \quad p_{RP2} < 17.2\%$), 5 points for achieving computationally desirable outcome (i.e., $p_{LC} > 50\% \quad {\text{and}} \quad p_{RP2} < 50\%$), and -1 point for failing to achieve a desirable outcome altogether. The negative point motivates the AI agent to search for the optimal dose as soon as possible.

### Wasserstein generative adversarial network with gradient penalty (WGAN-GP)

To compensate for low number of data points we employed WGAN-GP^43^, which learns the underlying data distribution and generates more data points. We generated 4000 additional data points for training qDRL models. Having a larger training dataset helps the reinforcement learning algorithm in accurately representing the state space. The training details are presented in the Supplementary Material.

## Supplementary Information

Supplementary Information.

## Data Availability

The training data analyzed in this study are obtained from the University of Michigan study UMCC 2007.123 (NCI clinical trial NCT01190527). Restrictions apply to the availability of these data, which were used under data sharing protocol for this study. The validation data analyzed in this study are obtained from the RTOG-0617 study (NCI clinical trial NCT00533949) and is publicly available in NCTN/NCORP Data Archive. Restrictions apply to the availability of these data, which were used under license for this study. Data are available from the authors upon reasonable request with the permission of NCI.

## Code availability

Software codes used in this study will be made available from the corresponding author on reasonable request.

## References

[1] Bryant AK (2017). Trends in radiation therapy among cancer survivors in the United States, 2000–2030. Cancer Epidemiol. Biomark. Prev..

[2] El Naqa I, Kosorok MR, Jin J, Mierzwa M, Ten Haken RK (2018). Prospects and challenges for clinical decision support in the era of big data. JCO Clin. Cancer Inform..

[3] Tseng H-H, Luo Y, Ten Haken RK, El Naqa I (2018). The role of machine learning in knowledge-based response-adapted radiotherapy. Front. Oncol..

[4] Sonke JJ, Belderbos J (2010). Adaptive radiotherapy for lung cancer. Semin. Radiat. Oncol..

[5] Morgan HE, Sher DJ (2020). Adaptive radiotherapy for head and neck cancer. Cancers Head Neck.

[6] Rodrigues G (2015). Definitive and adjuvant radiotherapy in locally advanced non-small cell lung cancer: An American Society for Radiation Oncology (ASTRO) evidence-based clinical practice guideline. Pract. Radiat. Oncol..

[7] LeCun Y, Bengio Y, Hinton G (2015). Deep learning. Nature.

[8] Alzubaidi L, Zhang J, Humaidi AJ (2021). Review of deep learning: Concepts, CNN architectures, challenges, applications, future directions. J. Big Data.

[9] Sutton RS, Barto AG (2018). Reinforcement Learning: An Introduction.

[10] Mnih V (2015). Human-level control through deep reinforcement learning. Nature.

[11] Silver D, Schrittwieser J, Simonyan K (2017). Mastering the game of Go without human knowledge. Nature.

[12] Tseng HH (2017). Deep reinforcement learning for automated radiation adaptation in lung cancer. Med. Phys..

[13] Luo Y (2018). A multiobjective Bayesian networks approach for joint prediction of tumor local control and radiation pneumonitis in nonsmall-cell lung cancer (NSCLC) for response-adapted radiotherapy. Med. Phys..

[14] Tversky A, Kahneman D (1974). Judgment under uncertainty: Heuristics and biases. Science.

[15] Lichtenstein S, Slovic P (1971). Reversals of preference between bids and choices in gambling decisions. J. Exp. Psychol..

[16] Busemeyer JR, Bruza P (2012). Quantum Models of Cognition and Decision.

[17] Savage LJ (1954). The Foundation of Statistics.

[18] Finetti B. De, in *International Encyclopedia of the Social Sciences*, (ed. D. E. Sills), **12**, 496–504. (Macmillan, 1968)

[19] Shafir E, Tversky A (1992). Thinking through uncertainty: Non-consequential reasoning and choice. Cogn. Psychol..

[20] Pothos EM, Busemeyer JR (2009). A quantum probability explanation for violations of ‘rational’ decision theory. Proc. Biol. Sci..

[21] Yukalov VI, Sornette D (2016). Quantum probability and quantum decision-making. Phil. Trans. R. Soc. A.

[22] Khrennikov A (2018). Quantum probability in decision making from quantum information representation of neuronal states. Sci. Rep..

[23] Dong D, Chen C, Li H, Tarn T-J (2008). Quantum reinforcement learning. IEEE Trans. Syst. Man Cybern. Part B Cybern..

[24] Dong D, Chen C, Chu J, Tarn T-J (2012). Robust quantum-inspired reinforcement learning for robot navigation. IEEE/ASME Trans. Mechatron..

[25] Li J-A (2020). Quantum reinforcement learning during human decision-making. Nat. Hum. Behav..

[26] Asfaw, A. *et al*. Learn Quantum Computation Using Qiskit, http://community.qiskit.org/textbook (2020).

[27] IBM Q team, IBM Q 16 Melbourne backend specification V2.3.3, Retrieved from https://quantum-computing.ibm.com. (2020).

[28] Bradley JD (2015). Standard-dose versus high-dose conformal radiotherapy with concurrent and consolidation carboplatin plus paclitaxel with or without cetuximab for patients with stage IIIA or IIIB non-small-cell lung cancer (RTOG 0617): A randomised, two-by-two factorial phase 3 study. Lancet Oncol..

[29] Netherton TJ, Cardenas CE, Rhee DJ, Court LE, Beadle BM (2021). The emergence of artificial intelligence within radiation oncology treatment planning. Oncology.

[30] El Naqa I (2021). Prospective clinical deployment of machine learning in radiation oncology. Nat. Rev. Clin. Oncol..

[31] Watkins, C. J. C. H. Learning from Delayed Rewards, PhD Thesis, King’s College, University of Cambridge, England (1989). http://www.cs.rhul.ac.uk/~chrisw/new_thesis.pdf

[32] van Hassekt, H., Guez, A. & Silver, D. Deep Reinforcement Learning with Double Q-learning. Preprint at. https://arxiv.org/abs/1509.06461 (2015).

[33] Grover, L. K. A fast quantum mechanical algorithm for database search, Preprint at https://arxiv.org/abs/quant-ph/9605043 (1996).

[34] Nielsen MA, Chuang IL (2010). Quantum Computation and Quantum Information.

[35] Gilliam, A., Pistoia, M. & Gonciulea, C. Optimizing Quantum Search Using a Generalized Version of Grover’s Algorithm, Preprint at https://arxiv.org/abs/2005.06468 (2020).

[36] Luster A, Unkeless J, Ravetch J (1985). γ-Interferon transcriptionally regulates an early-response gene containing homology to platelet proteins. Nature.

[37] Dufour JH (2002). IFN-gamma-inducible protein 10 (IP-10; CXCL10)-deficient mice reveal a role for IP-10 in effector T cell generation and tracking. J. Immunol..

[38] Angiolillo AL (1995). Human interferon-inducible protein 10 is a potent inhibitor of angiogenesis in vivo. J. Exp. Med..

[39] Vallieres, M. C. Radiomics: enabling factors towards precision medicine, Phd Thesis, McGill University at https://escholarship.mcgill.ca/concern/theses/4f16c513z (2018).

[40] El Naqa I (2018). A Guide to Outcome Modeling in Radiotherapy and Oncology: Listening to the Data.

[41] Hildebrandt MAT (2010). Genetic variants in inflammation-related genes are associated with radiation-induced toxicity following treatment for non-small cell lung cancer. PLoS ONE.

[42] Borghesi, A., Baldo, F., & Milano, M. Improving Deep Learning Models via Constraint-Based Domain Knowledge: A Brief Survey, Preprint at https://arxiv.org/abs/2005.10691 (2020).

[43] Gulrajani, I. *et al*. Improved Training Wasserstein GANs, Preprint at https://arxiv.org/abs/1704.00028 (2017).

