# Author Correction: Quantum deep reinforcement learning for clinical decision support in oncology: application to adaptive radiotherapy

**DOI:** 10.1038/s41598-023-28810-x

**Published:** 2023-02-09

**Authors:** Dipesh Niraula, Jamalina Jamaluddin, Martha M. Matuszak, Randall K. Ten Haken, Issam El Naqa

**Affiliations:** 1 Department of Machine Learning, H. Lee Moffitt Cancer Center and Research Institute, Tampa, FL 33612 USA; 2 Department of Nuclear Engineering and Radiological Sciences, University of Michigan, Ann Arbor, MI 48109 USA; 3 Department of Radiation Oncology, University of Michigan, Ann Arbor, MI 48109 USA

Correction to: *Scientific Reports* 10.1038/s41598-021-02910-y, published online 07 December 2021

The Acknowledgements section in the original version of this Article was incomplete.

“This work was partially supported by the National Institute of Health (NIH) under Grant No. R01-CA233487. I.E.N. acknowledges support form NIH under Grant Nos. R41CA243722 and R37CA222215, and support from National Institute of Biomedical Imaging and Bioengineering under Contract No. 75N92020D00018. M.M.M acknowledges support in the form of research grant from Varian Medical Systems and is the co-director of Michigan Radiation Oncology Quality Consortium (Funded by Blue Cross Blue Shield Michigan). We acknowledge the use of the IBM Q for this work. The views expressed are those of the authors and do not reflect the official policy or position of IBM or the IBM Q team.”

now reads:

“This work was partially supported by the National Institute of Health (NIH) under Grant No. R01-CA233487. I.E.N. acknowledges support form NIH under Grant Nos. R41CA243722 and R37CA222215, and support from National Institute of Biomedical Imaging and Bioengineering under Contract No. 75N92020D00018. M.M.M acknowledges support in the form of research grant from Varian Medical Systems and is the co-director of Michigan Radiation Oncology Quality Consortium (Funded by Blue Cross Blue Shield Michigan). We acknowledge the use of the IBM Q for this work. The views expressed are those of the authors and do not reflect the official policy or position of IBM or the IBM Q team.

The authors also acknowledge that the RTOG0617 dataset was made available by NCT00533949-D1 from the NCTN Data Archive of the National Cancer Institute’s (NCI’s) National Clinical Trials Network (NCTN). Data were originally collected from clinical trial NCT number NCT00533949 [A Randomized Phase III Comparison of Standard- Dose (60 Gy) versus High-Dose (74 Gy) Conformal Radiotherapy with Concurrent and Consolidation Carboplatin/Paclitaxel + / − Cetuximab (Ind #103,444) in Patients with Stage IIIA/IIIB Non-Small Cell Lung Cancer]. All analyses and conclusions in this manuscript are the sole responsibility of the authors and do not necessarily reflect the opinions or views of the clinical trial investigators, the NCTN, or the NCI."

The original Article has been corrected.

