# Supplementary material for: Quantum deep reinforcement learning for clinical decision support in oncology: application to adaptive radiotherapy
Source: Sci Rep. 2021 Dec 7;11:23545. doi: 10.1038/s41598-021-02910-y (PMC8651664; doi:10.1038/s41598-021-02910-y)
Supplement: Supplementary file 1 — Supplementary Information. [file 41598_2021_2910_MOESM1_ESM.pdf]

Supplementary Material –
Quantum Deep Reinforcement Learning for Clinical Decision Support in Oncology: Application to Adaptive Radiotherapy

Dipesh Niraula[1], Jamalina Jamaluddin[2], Martha M. Matuszak[2,3], Randall K. Ten Haken[3], Issam El Naqa[1]

[1]Department of Machine Learning, H. Lee Moffitt Cancer Center & Research Institute, Tampa, FL 33612, USA.

[2]Department of Nuclear Engineering & Radiological Sciences, University of Michigan, Ann Arbor, MI 48109, USA.

[3]Department of Radiation Oncology, University of Michigan, Ann Arbor, MI 48109, USA.

Contents

1 Quantum Controller Circuit 2

2 Transition Function Neural Network (TF-NN) 3

3 Transition function for Generalized Equivalent Uniform Dose (gEUD) 4

4 Radiotherapy Outcome Estimator 5

5 Training Dataset 7

6 Validation Dataset 7

7 Wasserstein Generative Adversarial Network with Gradient Penalty (WGAN-GP) 9

8 Deep Q-Net Architecture 12

Reference 19

1 Quantum Controller Circuit

The quantum deep reinforcement learning (qDRL) algorithm was run on the 15-qubit IBM Quantum processor, IBMQ 16 Melbourne [1]. Because IBMQ is an open access system and, as IBMQ 16 Melbourne is the only 15-qubit computer, the wait time was excessively long, making it impossible to complete the QRL algorithm in a reasonable time. Due to the long waiting time, we divided the qDRL training into two parts: quantum decision-making and updating of the deep q-net. All the quantum computation were conducted in Qiskit quantum computing package [2] including connecting to the IBMQ quantum processor.

We chose a 5-qubit quantum system which can represent 32 decision state. For this study, we selected the dose decision ranging from 1.5 to 4 Gy/frac divided in 32 equal intervals. We designed 32 quantum controller circuits representing each dose decision. We then ran a sufficiently large number of experiments for all 32 controller circuits in the quantum processor, stored the results locally, and later used them for qDRL training. Specifically, we ran 8000 experiments (shots) for every controller circuit and then repeated 20 different trials for each controller circuit. Here the distinction between experiment and trial is that experiments are repeated measurement on a controller circuit while trial corresponds to setting up a new but identical controller circuit before running the experiments. This was done to ensure that we could average out any noise beside for the quantum noise. So, for the case of 5 qubits, a total of 5,120,000 experiments (32 states $\times$ 20 trials $\times$ 8000 shots) were carried out, which took about a week for its completion. Then, for each decision selection process (quantum amplification + measurement) during the qDRL training episode, a random trial was chosen, and then a random experiment was picked corresponding to the optimal decision state.

2 Transition Function Neural Network (TF-NN)

The architectures of TF-NN are given in Table 1. Individual TF-NN maps the 5×1 dimensional feature variable and the 1×1 dimensional dose-decision (dose/frac) to the 1×1 dimensional single feature variable of the next state. Note, we have added an extra layer (preprocess) that maps the 1×1 dimensional dose-decision to the 5×1 dimensional hidden layer before being concatenated with the 5×1 dimensional feature variable and before feeding into the NN. This is done so that dose-decisions and states can have equal footing, as radiation dose are as important as state. We used Mean Square Error (MSE) as the loss function and Adam with a learning rate of 5×10^{-4} as the optimizer. To avoid overfitting, we added a 20% random dropout layer, and trained the NN for a relatively small 500 training epochs. We standardized (z-score) the dataset for training and used Pytorch deep learning library [3].

TABLE 1. Transition Function Neural Network Architecture

Layer Name	Formula	Dimension
Input	(s, d)	$s:\ 5 \times 1;\ d : 1 \times 1$
Preprocess	$\boldsymbol{x} = \text{concat}(s, (w_d . d + b_d))$	$w_d :\ 5 \times 1;\ b_a : 5 \times 1;\ x : 10 \times 1$
Hidden Layer 1	$z_1 = \text{elu}(w_1 . x + b_1)$	$w_1 :\ 128 \times 10;\ b_1 : 128 \times 1;\ z_1 : 128 \times 1$
Regularization 1	$r_1 = 20\%$ dropout in z_1	$r_1 :\ 128 \times 1$
Hidden Layer 2	$z_2 = \text{elu}(w_2 . r_1 + b_2)$	$w_2 :\ 128 \times 128;\ b_2 : 128 \times 1;\ z_2 : 128 \times 1$
Regularization 2	$r_2 = 20\%$ dropout in z_2	$r_2 : 128 \times 1$
Hidden Layer 3	$z_3 = \text{elu}(w_3 . r_2 + b_3)$	$w_3 :\ 128 \times 128;\ b_3 : 128 \times 1;\ z_3 : 128 \times 1$
Regularization 3	$r_3 = 20\%$ dropout in z_3	$r_3 :\ 128 \times 1$
Output	$s' = w_o . r_3 + b_o$	$w_o :\ 1 \times 128;\ b_o : 1 \times 1;\ s' : 1 \times 1$

Abbreviation: concat, Concatenate; elu, exponential linear unit

3 Transition function for Generalized Equivalent Uniform Dose (gEUD)

To maintain the monotonic relation between the gEUD and dose fractionation we replaced NN by a function of the form,

$$\frac{g(t_n) - g(t_{n-1})}{\Delta t} \propto d_n \left(1 + \frac{d_n}{\frac{\alpha}{\beta}} \right), \mathrm{n} \in \mathbb{N} - (1)$$

given that same number of dose fractionations are administered in Δt. Here, the time is divided into n intevals, so we rewrite $g(t_n)$ as g_n. We know that gEUD before starting the treatment has to be zero, i.e., $g_0 = 0 \text{ for } t_0$; taking this fact into consideration and starting from the assumption (1) we obtain the following relationship,

$$g_n = g_{n-1} + g_1 \frac{d_n \left(\frac{\alpha}{\beta} + d_n \right)}{d_1 \left(\frac{\alpha}{\beta} + d_1 \right)} - (2).$$

This relationsip tells if we know the ratio between g_1 and $d_1 \left(\frac{\alpha}{\beta} + d_1 \right)$ for a patient (i.e., the gEUD at week 2 for dose fractionation d_1), then we can extrapolate g_n for a patient with known g_{n-1} gEUD who will be administrated d_n dose. An example of Eq. (2) is given in Figure 1.

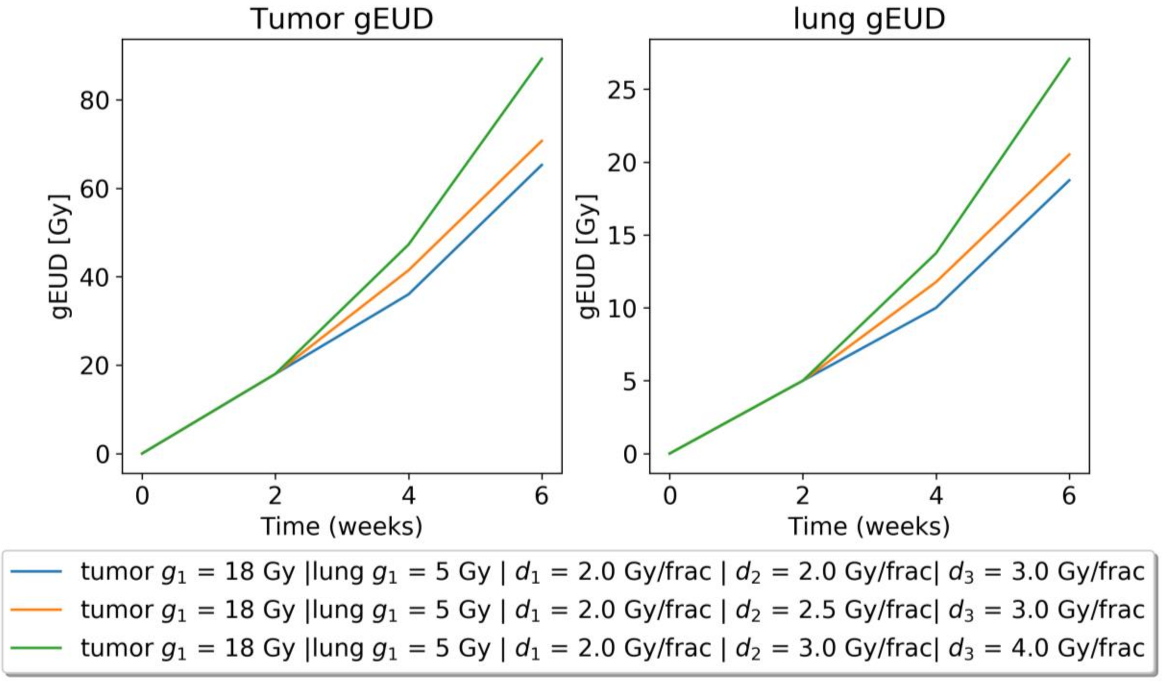

Figure 1. Tumor and lung gEUD transition function.

Plots showing time transition of tumor and lung gEUDs for three different scenarios. Here α/β ratios are 10 and 4 for tumor and lung gEUDs, respectively.

4 Radiotherapy Outcome Estimator

The architectures of NN classifiers used as the RT outcome estimator are given in Table 2. LC classifier maps tumor gEUD, cxcr1-Rs2234671, and GLSZM-ZSV to LC outcome, and RP2 classifier maps lung gEUD, cxcr1-Rs2234671, and IP10 to RP2 outcome. We trained the classifiers for 300 training epochs. We applied Xavier Normalization to the initial weights, binary cross entropy (BCE) as loss function, Adam with a learning rate of 5×10^{-4} as the optimizer for learning μ and 0.1×10^{-4} for learning T. To force a monotonic relationship between radiation dose and RT outcome and maintain the probability measure we used a generic logistic function as shown in Table 2. Training of NNs for μ and T were carried out sequentially i.e., training one NN while leaving the other NN frozen.

TABLE 2. RT outcome estimator Neural Network Architecture

Layer Name	Formula	Dimension
Input	s', g'	$s': 2 \times 1;\ g': 1 \times 1$
	NN1 for $\boldsymbol{\mu}$	
Hidden Layer 1	$z_1 = \mathrm{elu}(w_1.s' + b_1)$	$w_1:\ 128 \times 2;\ b_1: 128 \times 1;\ z_1: 128 \times 1$
Hidden Layer 2	$z_2 = \mathrm{elu}(w_2.z_1 + b_2)$	$w_2:\ 128 \times 128;\ b_2: 128 \times 1;\ z_2: 128 \times 1$
Hidden Layer 3	$z_3 = \mathrm{elu}(w_3.z_2 + b_3)$	$w_3:\ 128 \times 128;\ b_3: 128 \times 1;\ z_3: 128 \times 1$
Meta-output 1	$\mu = \mathrm{sigmoid}(w_o.z_3 + b_o)$	$w_o:\ 1 \times 128;\ b_o: 1 \times 1;\ \mu: 1 \times 1$
	NN2 for $\boldsymbol{T}$	
Hidden Layer 1	$\boldsymbol{z_1 = \mathrm{elu}(w_1.s' + b_1)}$	$\boldsymbol{w_1:\ 128 \times 2;\ b_1: 128 \times 1;\ z_1: 128 \times 1}$
Hidden Layer 2	$z_2 = \mathrm{elu}(w_2.z_1 + b_2)$	$w_2:\ 128 \times 128;\ b_2: 128 \times 1;\ z_2: 128 \times 1$
Hidden Layer 3	$z_3 = \mathrm{elu}(w_3.z_2 + b_3)$	$w_3:\ 128 \times 128;\ b_3: 128 \times 1;\ z_3: 128 \times 1$
Meta-output 2	$T = \mathrm{sigmoid}(w_o.z_3 + b_o)$	$w_o:\ 1 \times 128;\ b_o: 1 \times 1;\ T': 1 \times 1$
	Merge NN1 and NN2	
Output	$p_{LC\|RP2} = \dfrac{1}{1 + \exp\left(\dfrac{g' - \mu}{T}\right)},$	

Note: $g'$is min-max normalized to (0,1) by the population extremum.

5 Training Dataset

Information on a total of 67 patients with stage III NSCLC treated in and before 2009 [4] were available from prospective clinical studies. Forty-seven cases showed LC and 17 cases showed RP2. All the patients were treated with conventional fractionated radiotherapy via 3D conformal techniques. As seen from Figure 5, about half of the patients were treated with standard doses of 60-66 Gy in 30 fractions while the other half of the patients participated in an adaptive dose escalation study, where the dose was intensified during treatment with 2.1-2.85 Gy/frac up to a total dose of 85.8 Gy in 30 fractions. All tumor and lung 3D dose value were converted to the standard 2Gy equivalents (EQD2) using the linear quadratic model and then generalized equivalent uniform doses (gEUDs) were calculated for gross tumor volumes according to the Table 2.

To learn the state dynamics as a function of the radiation, the dataset was divided into 3 time-steps in a 2-week interval: weeks 0, 2, and 4. Transition functions were trained with concatenated weeks 0 and 2 data as input (including dose fractionation) and weeks 2 and 4 data as output. A trained transition function could estimate patient's states 2 weeks into the future. RT outcome estimator were then trained with the estimated week 6 state as input and clinical treatment outcome as output. At last, the qDRL was trained with the week-4 patient data including the 4000 WGAN-GP generated dataset.

6 Validation Dataset

Information on a total of 174 patients with NSCLC and treated under RTOG 0617 protocol [5] were available for external validation. Out of the 544 total patients, 287 patients were treated with chemoradiotherapy only and remaining were treated with chemoradiotherapy plus cetuximab. Out of the 287 only 174 patients had their 3D dose distribution map available, out of which 96 patients received 60 Gy of radiation in 30 fractions and the remaining received 74 Gy of radiation in 37 fractions. For the latter case, we estimated the biologically equivalent dose (BED) fractionation value for the last two weeks of the treatment phase by converting the 37 (20 + 17) fractions into 30 (20 +10) fractions. We calculated 3.11 Gy/frac for the last 2 weeks.

Since the validation datasets were obtained from different protocols than the training datasets, we had to make several adjustments to our model for the validation purpose. First, information on cytokines, SNPs, and radiomics information were not available. Since amount of radiation received by the lung and tumor tissue are the strongest predictor of RT outcomes, we imputed

our model to be a function of only the lung and tumor gEUD. However, the μ and T parameters of the RT outcome estimator were taken from averaging, over training population, the outcome estimation that was trained with all 5 features as shown in Figure 2. The values of μ and T for LC were 55.41 ± 26.53 Gy and 30.95 ± 26.13 Gy and for RP2 and were 18.31 ± 9.13 Gy and 7.37 ± 6.80 Gy, respectively.

Additionally, instead of RP2, the RTOG dataset contained a more severe grade 3 radiation-induced pneumonitis as outcome. We relied on following two assumptions for adapting our self-evaluation schemes for the analysis: (1) patients who develop RP3, develop RP2 as well, and (2) patients that are more likely to have RP2, are also more likely to have RP3.

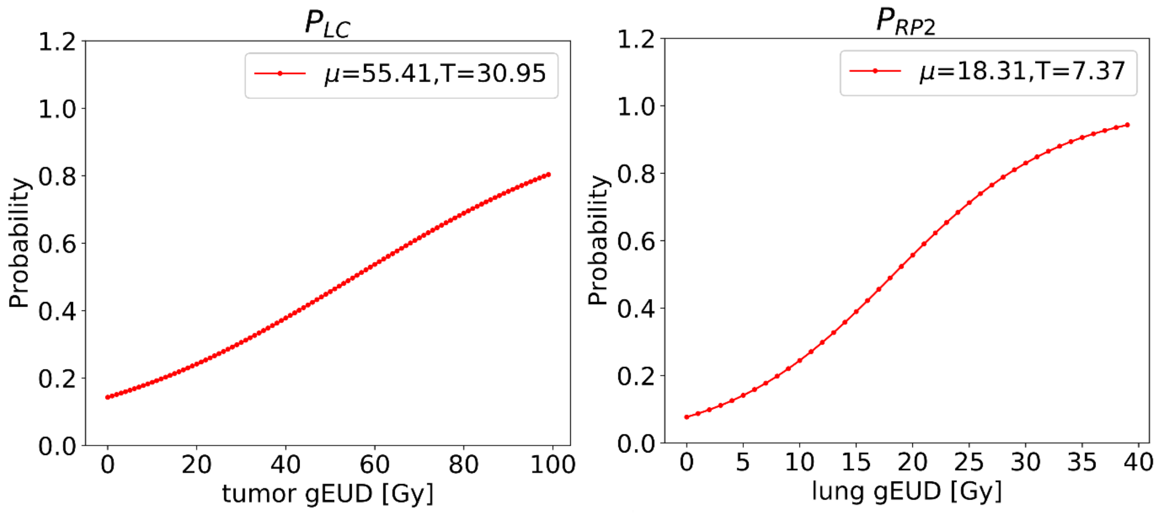

Figure 2: Population average RT outcome estimation for validation purpose.

Sigmoid-shaped probability estimation for LC and RP2 RT outcome as a function of tumor and lung gEUD obtained from averaging over the training population. The averaging was done for the RT outcome estimation that was trained using all 5 features. The above logistic functions were used as the RT outcome estimator for training the qDRL for external validation.

7 Wasserstein Generative Adversarial Network with Gradient Penalty (WGAN-GP)

Due to the small number of datasets, we used WGAN-GP to learn the underlying data distribution and generate additional datapoints. We used 4000 generated datapoints for training the reinforcement learning models, which is necessary for learning an adequate state representation. The architecture of WGAN is given in Table 3. All the weights were randomly initialized with normal distribution of 0.05 standard deviation. The training and data generation was carried out via Tensorflow Keras deep learning library [6]. We used Adam optimizer with 10^{-4} learning rate, 0.5 β_1, and 0.9 β_2 as hyperparameter. For additional validation, we calculated the Jensen Shannon Divergence metric (JSD) between the original and the generated data distribution. The comparison is presented in Figure 3. Note, 0 JSD means complete overlap of two distributions and 1 JSD means complete separation.

TABLE 3. WGAN Architecture

Layer Name	Formula	Dimension
	Generator	
Input	n	n: 64×1
Hidden Layer 1	$z_1 = \text{elu}(w_1.n + b_1)$	w_1 : 128×2; b_1 : 128×1; z_1 : 128×1
Hidden Layer 2	$z_2 = \text{elu}(w_2.z_1 + b_2)$	w_2 : 128×128; b_2 : 128×1; z_2 : 128×1
Hidden Layer 3	$z_3 = \text{elu}(w_3.z_2 + b_3)$	w_3 : 128×128; b_3 : 128×1; z_3 : 128×1
Hidden Layer 4	$z_4 = \text{elu}(w_4.z_3 + b_4)$	w_4 : 128×128; b_4 : 128×1; z_4 : 128×1
Output	$g = w_o.z_3 + b_o$	w_o : 5×128; b_o : 5×1; g : 5×1
	Discriminator	
Input	x	x : 5×1
Hidden Layer 1	$z_1 = \text{elu}(w_1.x + b_1)$	w_1 : 128×5; b_1 : 128×1; z_1 : 128×1
Hidden Layer 2	$z_2 = \text{elu}(w_2.z_1 + b_2)$	w_2 : 128×128; b_2 : 128×1; z_2 : 128×1
Hidden Layer 3	$z_3 = \text{elu}(w_3.z_2 + b_3)$	w_3 : 128×128; b_3 : 128×1; z_3 : 128×1
Hidden Layer 4	$z_4 = \text{elu}(w_4.z_3 + b_4)$	w_4 : 128×128; b_4 : 128×1; z_4 : 128×1
Output	$d = w_o.z_3 + b_o$	w_o : 1×128; b_o : 1×1; d : 1×1

Here n is the random noise and x is the input to the discriminator which can either be real patient state s or generated state g.

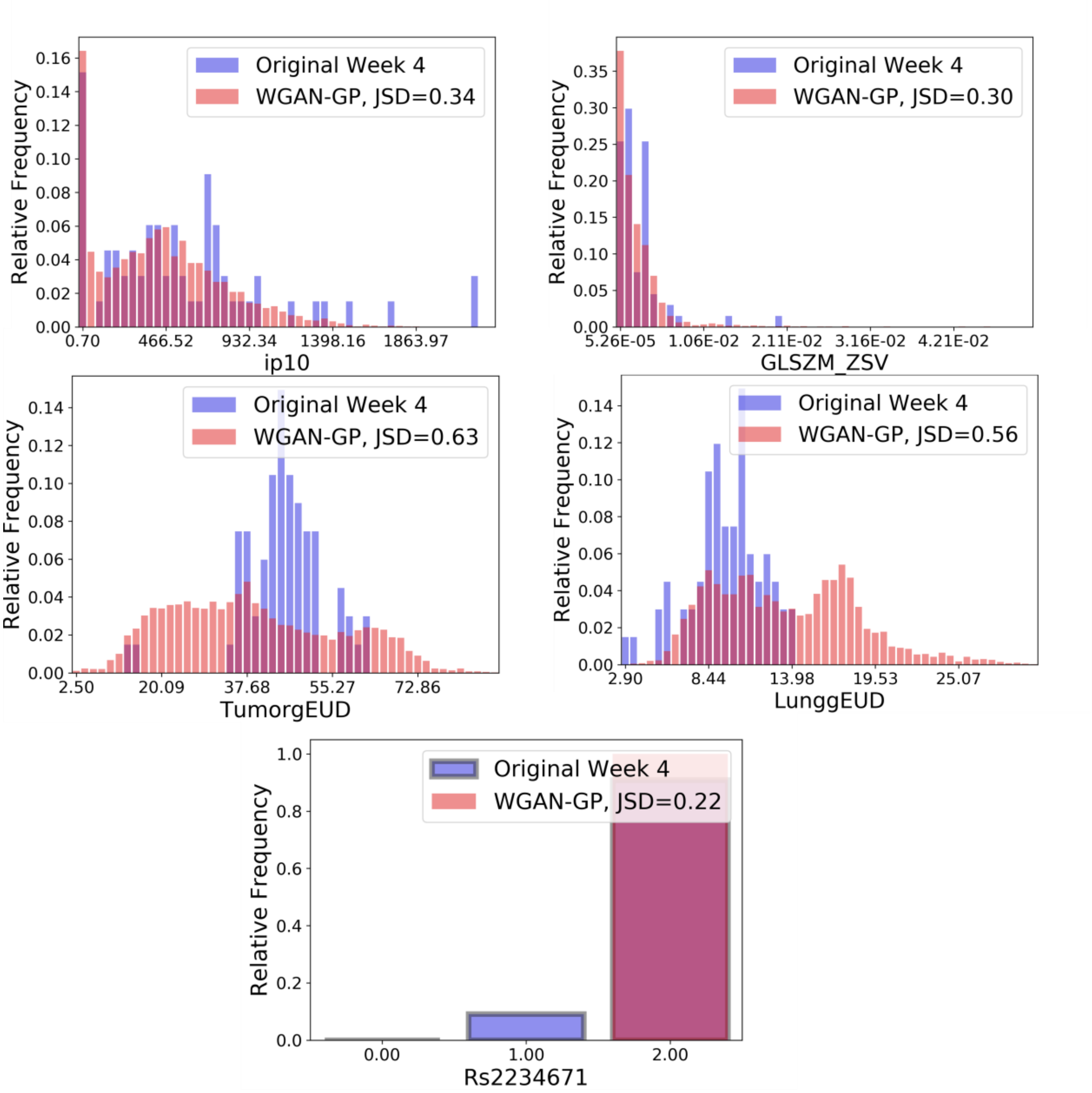

Figure 3. Wasserstein Generative Adversarial Network with Gradient Penalty generated dataset.

Comparison of data distribution between original and generated dataset. WGAN-GP was applied to generate week 4 dataset for training Deep Q-net. For quantitative comparison, Jensen Shannon Divergence metrics is calculated and presented in the figure.

8 Deep Q-Net Architecture

We applied double Q-learning algorithm for our framework, which uses two neural networks, namely policy net and target net. This approach helps in correcting for overestimating of the q-values [7]. The architecture of deep q-net is presented in Table 4. For training parameters, we set batch size to 64, gamma (discount factor) to 0.8, polyak factor to 0.999 (used for updating the target net's parameter) and Adam optimizer's learning rate to 10^{-4}. For DRL model, we used Boltzmann schema for balancing exploration-exploitation dilemma, where we set the starting epsilon to 0.95, ending epsilon to 0.1, and epsilon decay to 50,000 steps. We trained all models for 50,000 episode and set the replay memory size as 5000. We set the terminating steps to 10 steps i.e., new episode automatically begins if the agent in unsuccessful in achieving at least the computationally desirable outcome after 10 steps. All trainings were carried out in DGX-A100 system. AI recommendations from all three trained models for patients in both the training and validation datasets are presented in Figure 4 to Figure 9.

TABLE 4. Deep Q-Net Architecture

Layer Name	Formula	Dimension
Input	s	$s:\ 5 \times 1$
Hidden Layer 1	$z_1 = \mathrm{elu}(w_1.x + b_1)$	$w_1:\ 128 \times 5;\ b_1: 128 \times 1;\ z_1: 128 \times 1$
Hidden Layer 2	$z_2 = \mathrm{elu}(w_2.z_1 + b_2)$	$w_2:\ 128 \times 128;\ b_2: 128 \times 1;\ z_2: 128 \times 1$
Hidden Layer 3	$z_3 = \mathrm{elu}(w_3.z_2 + b_3)$	$w_3:\ 128 \times 128;\ b_3: 128 \times 1;\ z_3: 128 \times 1$
Hidden Layer 4	$z_4 = \mathrm{elu}(w_4.z_3 + b_4)$	$w_4:\ 128 \times 128;\ b_4: 128 \times 1;\ z_4: 128 \times 1$
Output	$q = w_o.z_3 + b_o$	$w_o:\ 32 \times 128;\ b_o: 32 \times 1;\ q: 32 \times 1$

Note: Since RTOG 0617 data only had lung and tumor gEUD, for validaiton purpose the input dimension is 2×1. Additionally, we also added week 2 gEUDs and dose fractionation as input feature, making the input dimension back to 5×1.

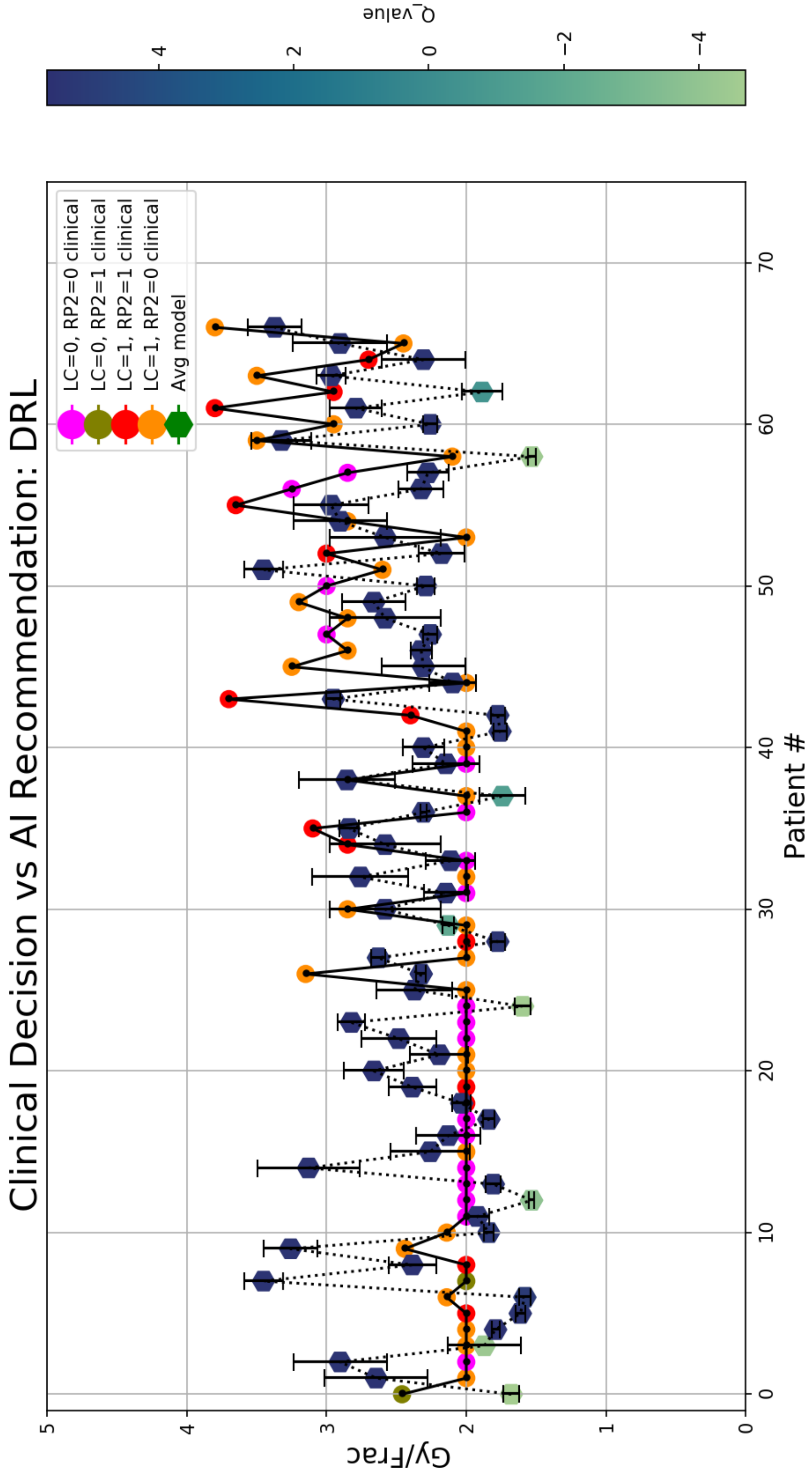

Figure 4. Training results for DRL.

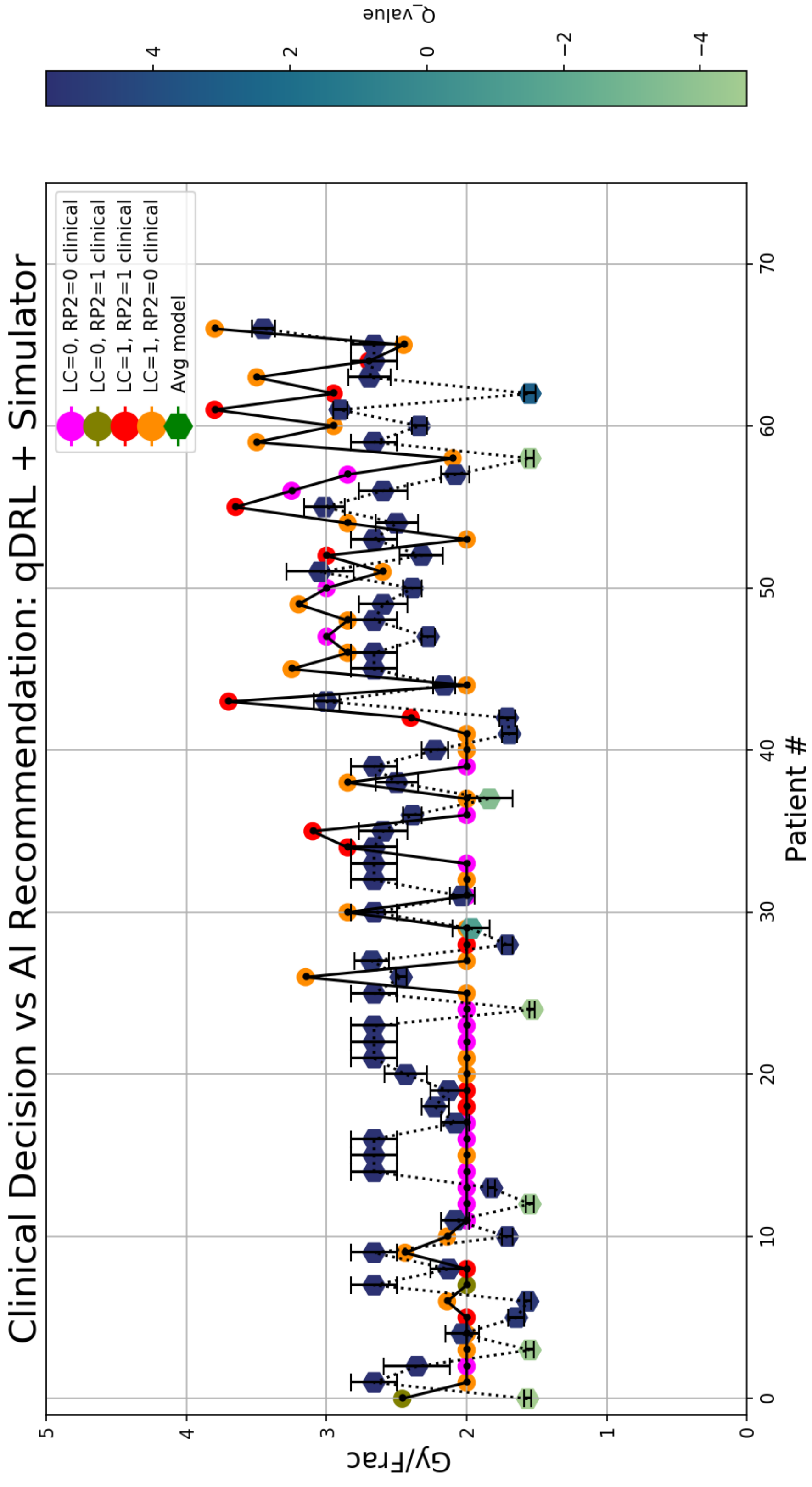

Figure 5. Training result for qDRL + simulator.

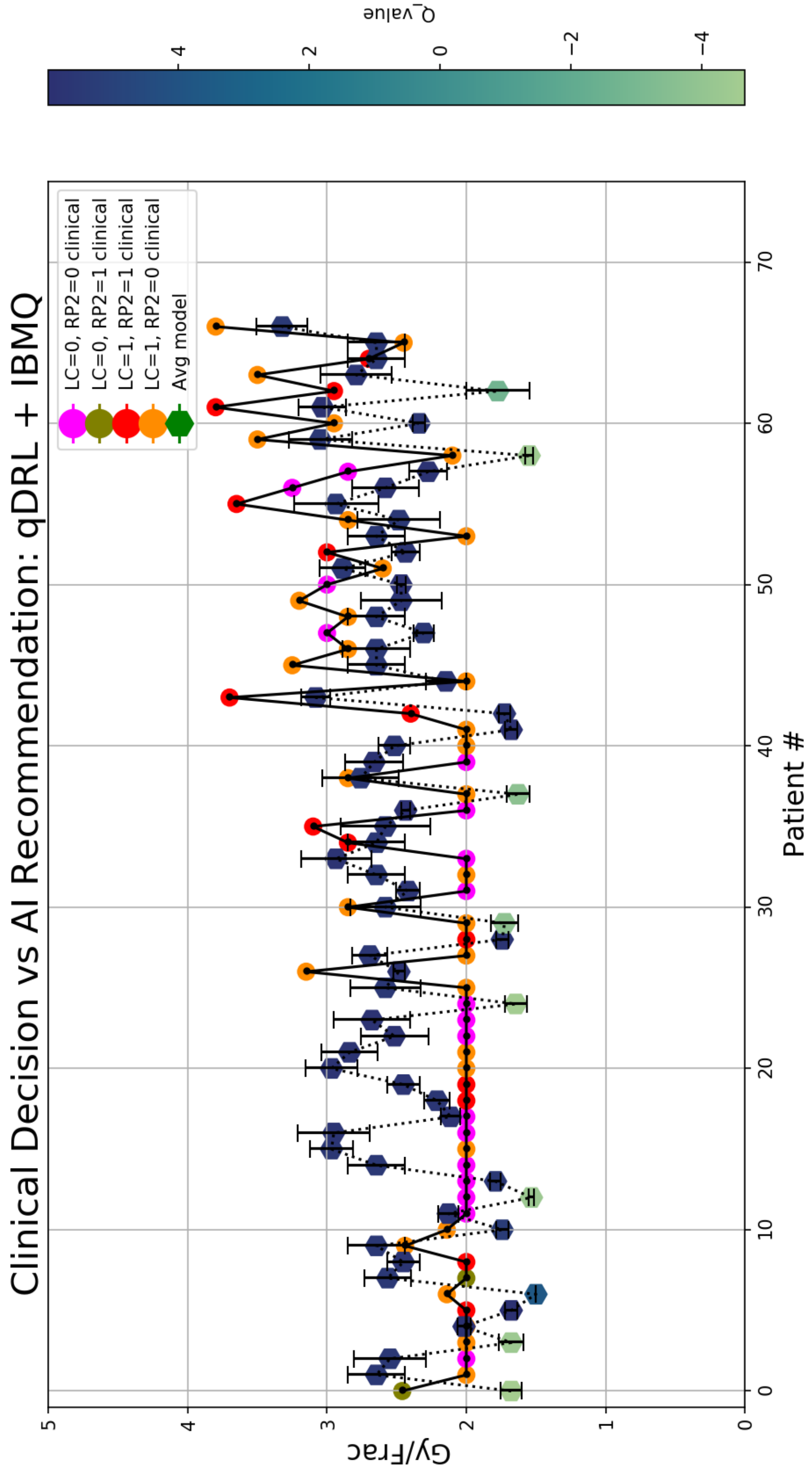

Figure 6. Training results for qDRL+ IBMQ.

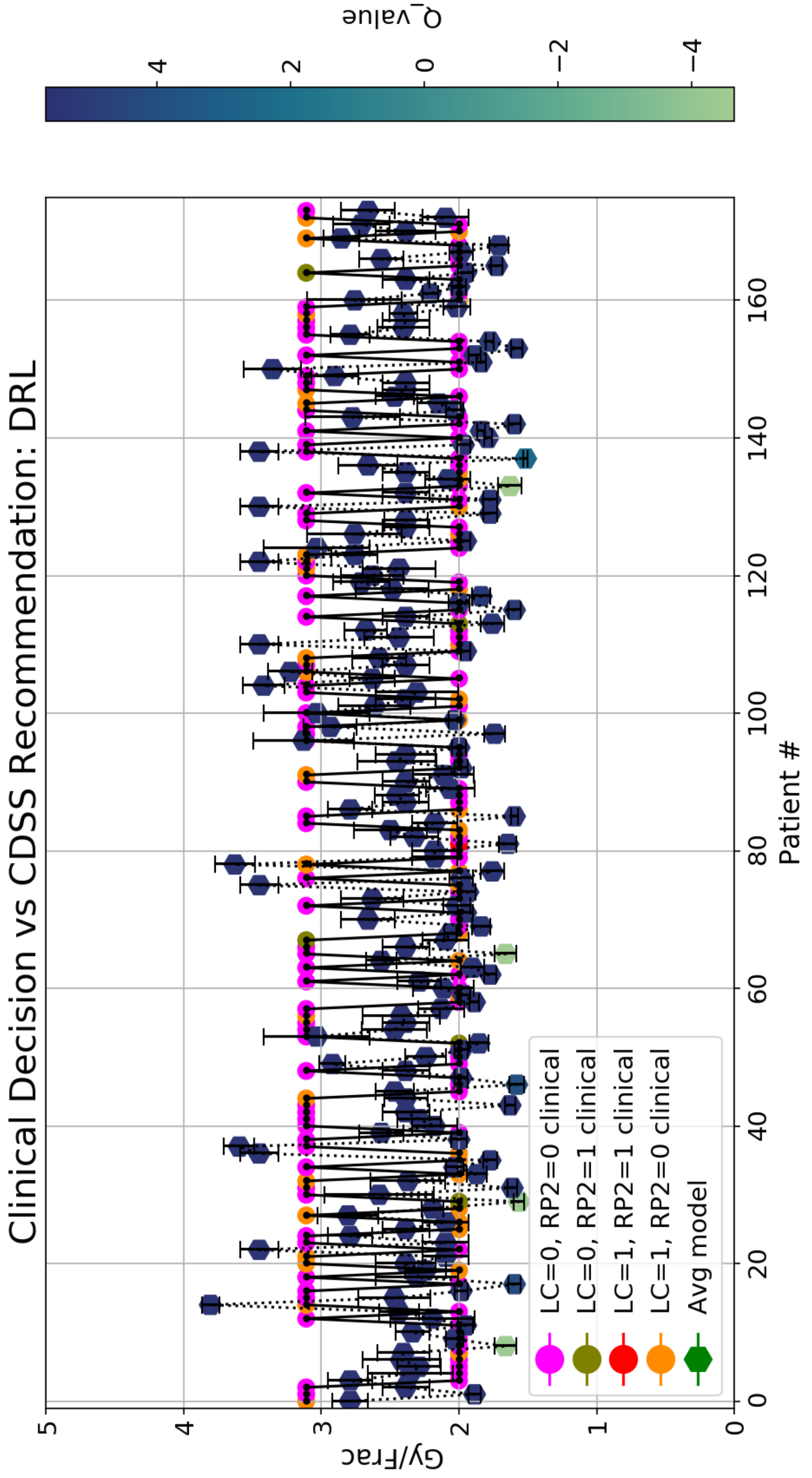

Figure 7. Validation result for DRL.

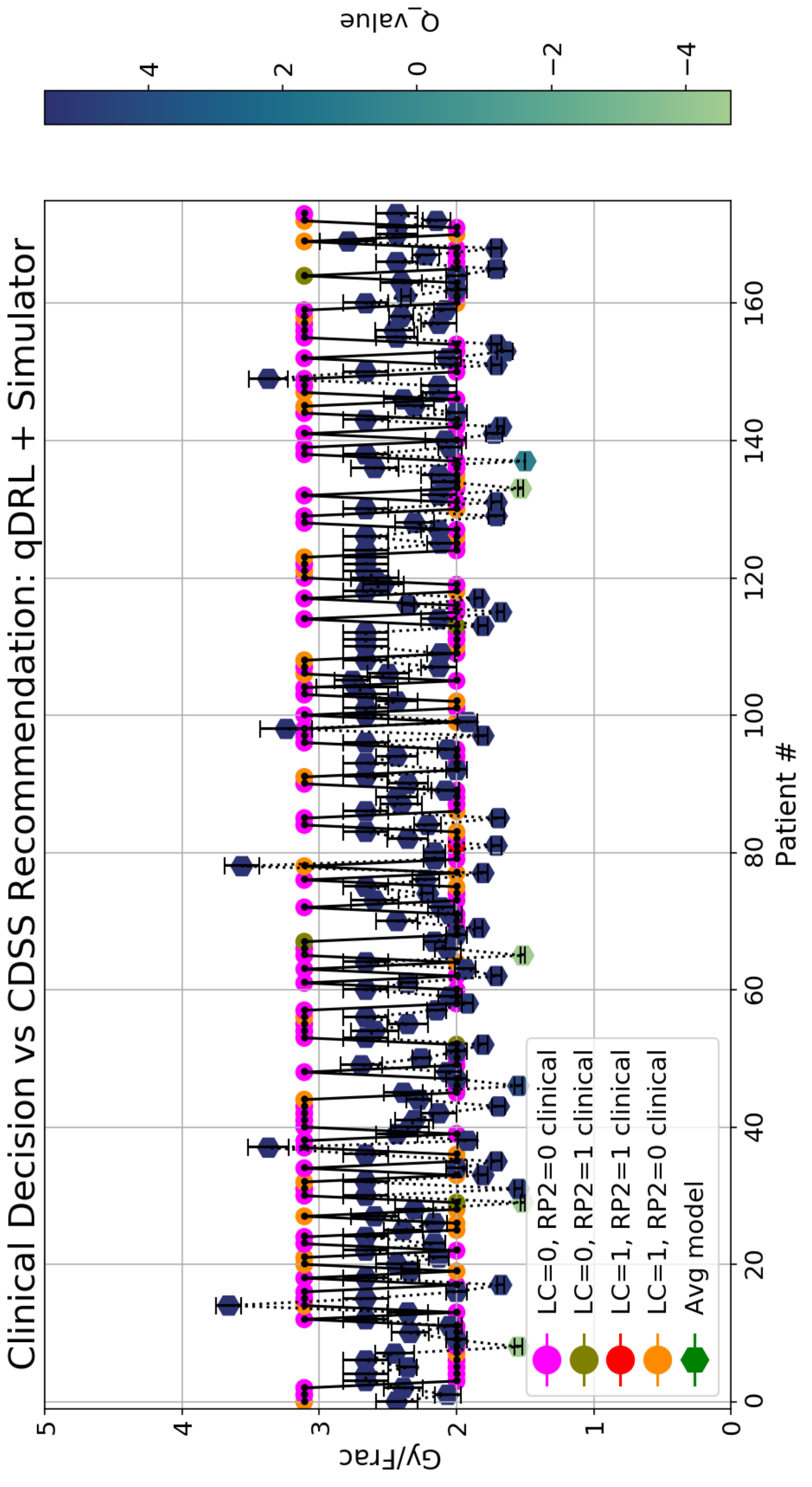

Figure 8. Validation result for qDRL + simulator.

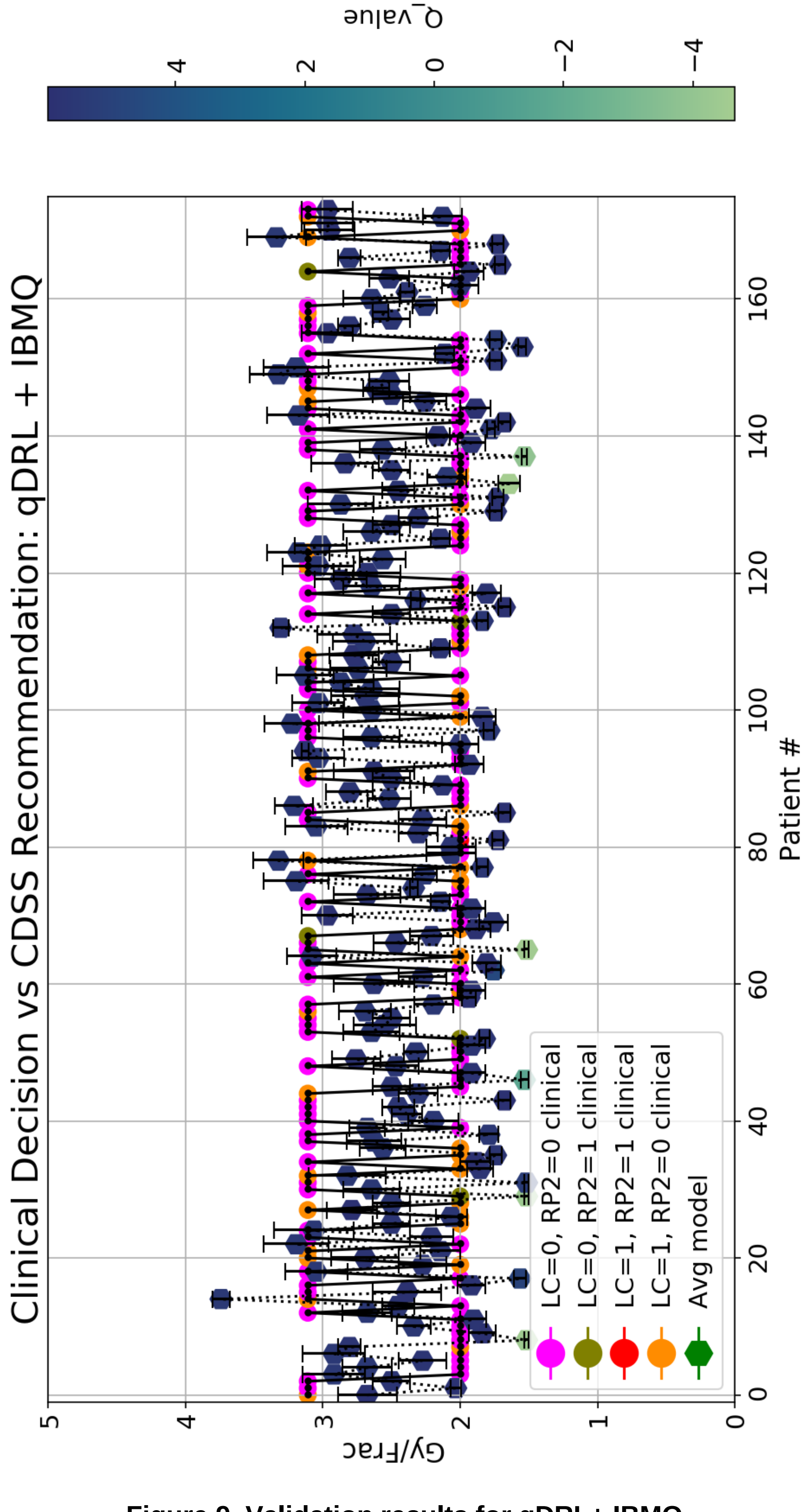

Figure 9. Validation results for qDRL+ IBMQ.

Reference

[1] IBM Q team, "IBM Q 16 Melbourne backend specification V2.3.3," (2020). Retrieved from https://quantum-computing.ibm.com.

[2] A. Asfaw, L. Bello, Y. Ben-Haim, et al., Learn Quantum Computation Using Qiskit, 2020. http://community.qiskit.org/textbook

[3] A. Paszke, S. Gross, F. Massa, et al., PyTorch: An Imperative Style, High-Performance Deep Learning Library, Advances in Neural Information Processing Systems 32, Curran Associates, Inc., pp. 8024-8035, 2019. http://papers.neurips.cc/paper/9015-pytorch-an-imperative-style-high-performance-deep-learning-library.pdf

[4] Y. Luo, D. L. McShan, M. M. Matuszak, et. al, A multiobjective Bayesian networks approach for joint prediction of tumor local control and radiation pneumonitis in nonsmall-cell lung cancer (NSCLC) for response-adapted radiotherapy, Med. Phys., vol. 45, no. 8, Aug 2018.

[5] J.D. Bradley, R. Paulus, R. Komaki, Standard-dose versus high-dose conformal radiotherapy with concurrent and consolidation carboplatin plus paclitaxel with or without cetuximab for patients with stage IIIA or IIIB non-small-cell lung cancer (RTOG 0617): a randomised, two-by-two factorial phase 3 study. Lancet Oncol. vol. 16, pp. 187–99. 2015 doi:10.1016/ s1470-2045(14)71207-0

[6] M. Abadi, A. Agarwal, P. Barham, et al., TensorFlow: Large-scale machine learning on heterogeneous systems, 2015. Software available from tensorflow.org. https://www.usenix.org/system/files/conference/osdi16/osdi16-abadi.pdf

[7] H. van Hassekt, A. Guez, and D. Silver, “Deep Reinforcement Learning with Double Q-learning,” arXiv:1509.06461v3 [cs.LG] 2015. https://arxiv.org/abs/1509.06461
